# Biophysical fitness landscape design traps viral evolution

**DOI:** 10.1101/2025.03.30.646233

**Published:** 2025-03-31

**Authors:** Vaibhav Mohanty, Eugene I. Shakhnovich

## Abstract

We introduce foundational principles for designing customizable fitness landscapes for proteins. We focus on crafting antibody ensembles to create evolutionary traps which restrict viral fitness enhancement. By deriving a fundamental relationship between a mutant protein’s fitness and its binding affinities to host receptors and antibodies, we show that the fitnesses of different protein sequences are designable, meaning they can be independently tuned by careful choice of antibodies. Given a user-defined target fitness landscape, stochastic optimization can be performed to obtain such an ensemble of antibodies which force the protein to evolve according to the designed target fitness landscape. We conduct *in silico* serial dilution experiments using microscopic chemical reaction dynamics to simulate viral evolution and validate the fitness landscape design. We then apply the design protocol to control the relative fitnesses of two SARS-CoV-2 neutral genotype networks while ensuring absolute fitness reduction. Finally, we introduce an iterative design protocol which consistently discovers better vaccination target sequences, generating antibodies that restrict the post-vaccination fitness growth of escape variants while simultaneously suppressing wildtype fitness. Biophysical fitness landscape design thus opens the door to prescient vaccine, antibody, and peptide design, thinking several steps ahead of pathogen evolution.

## 1 Introduction

Viruses constantly face pressure from human adaptive immunity to mutate, allowing the emergence of escape variants capable of increased replication and transmission, ultimately causing a greater number of human infections and deaths [1–4]. Even if vaccination efforts are rapid—like the development of the first SARS-CoV-2 mRNA vaccine [5]—by the time vaccines are adequately and widely deployed, viral escape mutations typcially will have already emerged, seeding yet another wave of infections. Current vaccine design thus always falls one step behind viral evolution, and waves of infection due to endemic viruses such as influenza exhibit a vicious cycle, requiring frequent and periodic updates to develop a new vaccine each year [1–4]. Developing an approach to break this cycle requires improved foresight into future viral evolution and adequate control over the viral fitness landscape to trap its evolutionary trajectory in a low fitness state.

Evolutionary fitness is a genotype's (or phenotype's) ability to reproduce, generally defined as a growth rate of a strain or species [6]. The relationship between the set of all genotypes and their respective fitnesses, often visualized as a mountain-like structure [7–10], is termed a fitness landscape. In the 90+ years since their introduction by Sewall Wright [11], fitness landscapes have proven to be a powerful tool for understanding evolutionary processes: namely, evolution can be viewed as a semi-random climb towards higher fitness peaks—a stochastic survival of the fittest [9, 12].

In this paper, we introduce foundational principles of biophysical *fitness landscape design* (FLD) to customize and shape the evolutionary landscape of evolving proteins. After deriving a biophysical model of viral fitness from microscopic chemical reactions, we show that careful choices of antibodies allow different viral surface protein mutants to be user-assigned different fitnesses with great flexibility and independence. Upon application of these antibodies, these genotypes then actually evolve according to the user-defined target landscape. We then introduce algorithms to design fitness landscapes appropriate to 2 application tasks: (1) molding the fitness landscape of two sets of SARS-CoV-2 neutral genotype networks and (2) discovery of vaccination targets which better restrict the fitness growth of viral escape variants compared to a typical vaccination protocol.

FLD opens the door to prescient vaccine and antibody design [13–18], with possible downstream applications to CAR-T cell therapy [19, 20] and small peptide drug design [21, 22]. More broadly, FLD brings delivers a major leap in addressing a fundamental open problem in evolutionary biology: controlling evolution to fight human disease.

## 2 Results

### 2.1 Antibody-antigen and host-antigen binding affinities determine viral fitness

In order to design fitness landscapes, we first obtain a biophysical model of fitness which has fully interpretable and tunable features. In this model, we consider a well-mixed solution of virions, antibodies, and host cells. We denote the viral strain as $\mathbf{s}$, the chemical species referring to the entire virion of that strain as $\mathrm{V}(\mathbf{s})$, and the chemical species referring to that strain's surface protein (fusion or spike) responsible for host cell entry as $\mathrm{V}_{\mathrm{ent}}(\mathbf{s})$. Similarly, the relevant subset of the antibody sequence (the paratope) is called $\mathbf{a}$, with the $n$-th type of antibody in the cocktail with paratope $\mathbf{a}_n$ called $\mathrm{Ab}_n(\mathbf{a}_n)$. Host cell receptors are called H, concentrations of any chemical species X are denoted by the usual brackets [X], and two bound chemical species X and Y are denoted as $\mathrm{X} \cdot \mathrm{Y}$.

The definition of absolute fitness $F(\mathbf{s})$ for a viral strain $\mathbf{s}$ is the baseline replication rate of the virus

$$\frac{\mathrm{d}[\mathrm{V}(\mathbf{s})]}{\mathrm{d}t} \equiv F(\mathbf{s})[\mathrm{V}(\mathbf{s})]. \tag{2.1}$$

Our model starts with three chemical reactions: the host-antigen binding reaction,

$$\mathrm{V}_{\mathrm{ent}}(\mathbf{s}) + \mathrm{H} \underset{K_{d,\mathrm{H}}(\mathbf{s})}{\rightleftharpoons} \mathrm{V}_{\mathrm{ent}}(\mathbf{s}) \cdot \mathrm{H}, \tag{2.2}$$

with dissociation constant $K_{d,\mathrm{H}}(\mathbf{s})$, the antibody-antigen binding reaction

$$\mathrm{V}_{\mathrm{ent}}(\mathbf{s}) + \mathrm{Ab}_n(\mathbf{a}_n) \underset{K_{d,\mathrm{Ab}}(\mathbf{s},\mathbf{a}_n)}{\rightleftharpoons} \mathrm{V}_{\mathrm{ent}}(\mathbf{s}) \cdot \mathrm{Ab}_n(\mathbf{a}_n), \tag{2.3}$$

with dissociation constant $K_{d,\mathrm{Ab}}(\mathbf{s},\mathbf{a}_n)$, and the replication reaction for a virion bound to $j$ host receptors

$$\mathrm{V}(\mathbf{s}) \cdot (j\,\mathrm{H}) \xrightarrow{j\,k_{\mathrm{rep}}} N_o\,\mathrm{V}(\mathbf{s}),. \tag{2.4}$$

where $k_{\mathrm{rep}}$ is the baseline rate of cell entry and replication via each of the $j$ receptors, and $N_o$ is the number of offspring produced. From these reactions, we calculate (see Methods for full derivation) that

$$\frac{\mathrm{d}[\mathrm{V}(\mathbf{s})]}{\mathrm{d}t} \approx k_{\mathrm{rep}}(N_o - 1)N_{\mathrm{ent}}p_b(\mathbf{s})[\mathrm{V}(\mathbf{s})], \tag{2.5}$$

where $N_{\mathrm{ent}}$ is the number of viral entry proteins on the surface, and $p_b(\mathbf{s})$ is the approximate fraction of viral entry proteins which are bound to host receptors at equilibrium:

$$p_b(\mathbf{s}) \approx \frac{[\mathrm{H}^{\mathrm{total}}]e^{-\beta\Delta G_{\mathrm{Ab}}(\mathbf{s})}}{C_0 + [\mathrm{H}^{\mathrm{total}}]e^{-\beta\Delta G_{\mathrm{H}}(\mathbf{s})} + \sum_n [\mathrm{Ab}_n^{\mathrm{total}}(\mathbf{a}_n)]e^{-\beta\Delta G_{\mathrm{Ab}}(\mathbf{s},\mathbf{a}_n)}}, \tag{2.6}$$

with $C_0$ being a reference concentration to set units, $\beta$ being inverse temperature, and $\Delta G_{\mathrm{H}}(\mathbf{s}) = \beta^{-1}\log K_{d,\mathrm{H}}(\mathbf{s})$ as well as $\Delta G_{\mathrm{Ab}}(\mathbf{s},\mathbf{a}_n) = \beta^{-1}\log K_{d,\mathrm{Ab}}(\mathbf{s},\mathbf{a}_n)$ being binding affinities. The result from eq. (2.5) is matched with the definition of absolute fitness in eq. (2.1) to provide a *free energy-fitness relation*:

$$F(\mathbf{s}) \approx k_{\mathrm{rep}}(N_o - 1)N_{\mathrm{ent}}p_b(\mathbf{s}). \tag{2.7}$$

For convenience—except where otherwise noted—any numerical values of fitness the rest of this paper will be scaled by $k_{\mathrm{rep}}(N_o - 1)N_{\mathrm{ent}}$ so fitness is normalized between 0 and 1. We now show how eq. (2.7) can be used to design fitness landscapes to shape evolutionary outcomes.

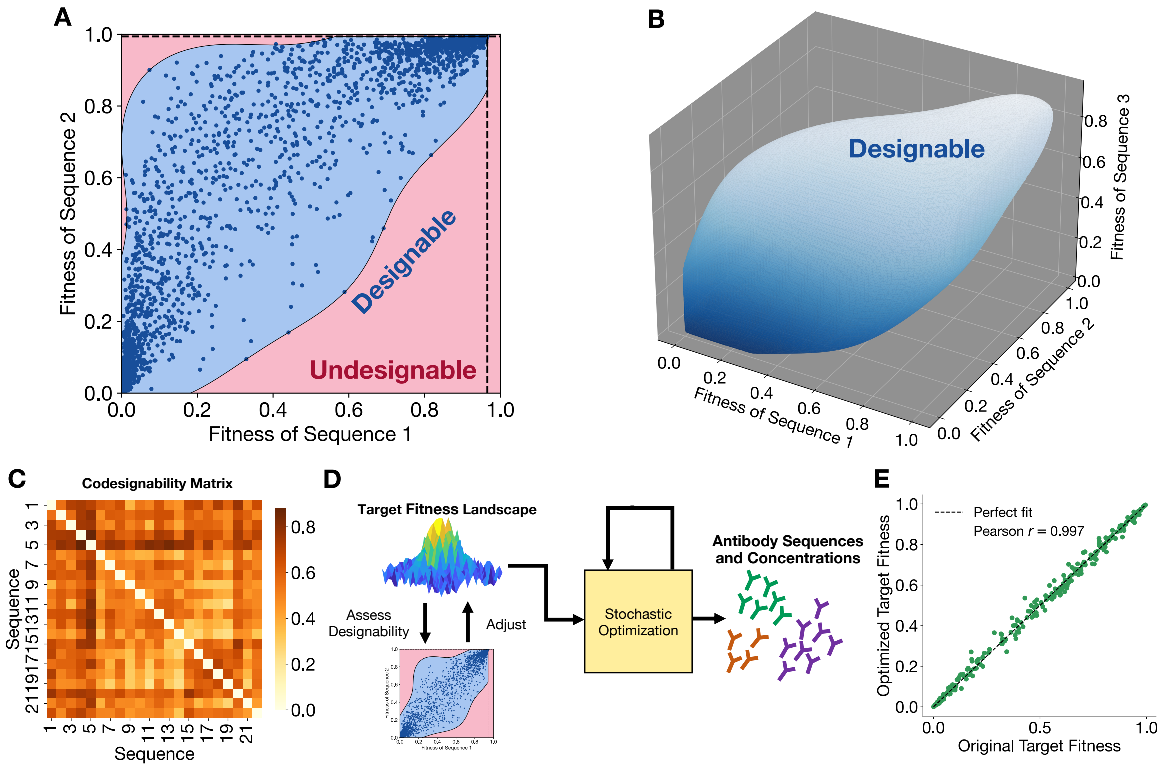

**Figure 1**: **Fitness landscapes are designable and can be custom designed with stochastic optimization.** (**A**) Designability phase diagram for two antigen sequences. Dark blue dots are randomly sampled fitness landscapes, each from a randomly generated antibody ensemble. The designable (light blue) region indicates possible fitness values that can be physically realized by some antibody ensemble. Black dashed lines indicate the maximum possible fitness for each sequence, limited by host-antigen binding affinity, according to eq. (2.7). (**B**) 3D designability phase diagram for three antigen sequences. (**C**) Codesignability matrix for a set of sequences, with higher codesignability scores indicating a larger designable region in the 2D phase diagram exmplified by (A). (**D**) Schematic for o-FLD protocol, which outputs the antibody sequences and concentrations which realize the target fitness landscape. (**E**) Near-exact agreement between target fitness landscape and the fitness landscape realized by antibodies discovered by the o-FLD protocol. *Note:* (**A**), (**B**), and (**E**) use normalized fitness, scaled down by $k_{\text{rep}}(N_o - 1)N_{\text{ent}}$.

### 2.2 Fitness landscapes are designable

To see how flexible or constrained the assignment of genotype-to-fitness really is, we used eq. (2.7) to explored the space of all possible fitness landscapes which could practically be produced by different choices of antibodies. To be concrete, we considered a set of mutable loci on the receptor binding domain (RBD) of the SARS-CoV-2 spike protein. We obtained Protein Data Bank (PDB) structures of the RBD bound to the human host receptor ACE2 which facilitates viral entry [23] and the RBD bound to Ly-CoV555, a class 2 antibody [24] which targets many of the same RBD amino acids which faciliate ACE2 binding [25, 26]. We allowed all 20 natural amino acid possibilities at 4 SARS-CoV-2 antigen sites and at 11 Ly-CoV555 paratope sites (Table S1).

We calculated fitness $F(\mathbf{s})$ for any antigenic variant $\mathbf{s}$ in the presence of any set of antibody variants $\{\mathbf{a}_n\}$ by using EvoEF force field calculations [27] for host-antigen binding free energies $\Delta G_{\mathrm{H}}(\mathbf{s})$ which were then calibrated to experimental results from ref. [28]. For antibody-antigen binding free energy prediction, a Potts model was trained [29, 30] on $\Delta G_{\mathrm{Ab}}(\mathbf{s}, \mathbf{a})$ force field calculations and similarly calibrated (see Methods for details). These binding free energies are plugged directly into eq. (2.7) to obtain fitness.

The key question of fitness landscape designability is: what fitnesses values are physically allowed to be assigned to genotypes? We first consider the simplest case of fitness landscape design: two randomly chosen antigen sequences, whose space of possible fitness landscapes is shown in Figure 1A. By generating random ensembles of antibodies (see Methods), we sampled the space of practically realizable fitness landscapes. We then used a support vector machine to obtain the decision boundary to separate the regions in which fitness pairs could exist, and where fitness pairs were not found. These two regions, respectively, are the *designable* and *undesignable* regions in Figure 1A, producing a "phase diagram" for fitness landscape designability.

Having a larger designable region (blue in Figure 1A) means that the user has increased flexibility in the assignment of fitnesses to different genotypes. The notion of designability and the designability phase diagram can certainly be generalized to more than 2 sequences (Figure 1B), but beyond 3 dimensions, visualizations become difficult. Instead, we can gather useful information about simultaneous design of fitness for many sequences by considering all possible pairs of sequences within a larger set. We introduce the *codesignability score*

$$C(\mathbf{s}_1, \mathbf{s}_2) = \frac{\text{designable region area for } \mathbf{s}_1 \text{ and } \mathbf{s}_2}{\text{designable + undesignable region areas}} \tag{2.8}$$

to accomplish this. The codesignability score $C(\mathbf{s}_1, \mathbf{s}_2)$ measures the blue (designable) fraction of the unit square in Figure 1A. For a set of sequences, we can visualize the codesignability between each pair of sequences in a codesignability matrix (Figure 1C). The darker the square, the higher the codesignability score, and the more flexibility in choice of fitnesses that can be realized by real antibody ensembles. We will show in a subsequent application that the codesignability matrix can capture useful biophysical information about neutral genotype networks.

#### 2.3 Designing fitness landscapes with stochastic optimization

After showing that there is great flexibility in assigning fitnesses to genotypes, we sought to obtain the antibody sequences and concentrations which, for a user-defined target fitness landscape $F_{\text{target}}(\mathbf{s})$, causes the viruses to actually experience that fitness landscape. We accomplish this task through stochastic optimization of a least squares loss function:

$$\mathcal{L}[F(\mathbf{s})|F_{\text{target}}(\mathbf{s})] \propto \sum_{\mathbf{s}} \left(F(\mathbf{s}) - F_{\text{target}}(\mathbf{s})\right)^2, \tag{2.9}$$

where $F(\mathbf{s})$ is given by the theory in eq. (2.7), and optimization takes place over antibody sequences and their concentrations. For a set of variants $\{\mathbf{s}\}$, we can first assess their designability by computing designability phase diagrams and/or the codesignability matrix. Then, given a target landscape $F_{\text{target}}(\mathbf{s})$, we perform simulated annealing [31] with the Metropolis-Hastings algorithm [32] to update antibody sequences and concentrations. The output of this optimization FLD (o-FLD) protocol (Figure 1D) is a list of antibody variants and their respective concentrations. We showcase the use of this o-FLD protocol to obtain an antibody ensemble for a random target fitness landscape chosen from the designable region for a set of 256 sequences. The o-FLD protocol is consistently successful, finding an ensemble of antibodies for which the model fitnesses $\{F(\mathbf{s})\}$ nearly perfectly match the target fitnesses $\{F_{\text{target}}(\mathbf{s})\}$ (Figure 1E). Thus, we have shown that fitness landscape design is indeed possible.

#### 2.4 *In silico* serial dilution experiments with chemical reaction dynamics validate designed fitness landscapes

After obtaining an antibody ensemble which theoretically should reproduce the target fitness landscape, we wanted to see if viral variants would actually evolve according to that target landscape. To validate FLD, we performed a series of *in silico* serial dilution experiments which allowed for strain-versus-strain competition between viral variants.

Using the BioNetGen biochemical reaction network modeling software [33], we simulated the time-dependent chemical reaction dynamics given by the microscopic reactions eq. (2.2), eq. (2.3), and eq. (4.7). For the 256 sequences for which we designed the fitness landscape, we conducted 255 *in silico* experiments. Labeling one sequence as the reference strain, each of the remaining 255 strains separately faced the reference strain in a head-to-head competition over the course of many broth dilutions. At each broth dilution step, the relative frequencies of the two viral strains were recorded, akin to sequencing *in vitro* samples. The *in silico* experiment is summarized by the schematic in Figure 2A. In each experiment, within a broth the two viral strains experienced exponential growth at different rates, as expected from eq. (2.5), causing the total viral count to form a prototypical sawtooth-like wave [34] over the course of many broth dilutions (Figure 2B).

To obtain external validation of the designed fitness landscapes, fitness estimation should be performed only on strain frequency data, recorded at each broth transfer step indicated by the gray dashed lines in Figure 2B. The time series of these recorded strain frequencies are shown in Figure 2C, representing the only observable data that an experimenter would have access to (i.e. no knowledge of the underlying chemistry). Using a simple equation derived in the Methods, the fitness difference $F(\mathbf{s}_{\text{test}}) - F(\mathbf{s}_{\text{ref}})$ between a test strain $\mathbf{s}_{\text{test}}$ and reference strain $\mathbf{s}_{\text{ref}}$ could be estimated from the frequency data in Figure 2C. Repeating the procedure for the remaining 254 test strains provided a complete observed fitness landscape as obtained from the *in silico* serial dilution experiments.

In Figure 2D, we plot the observed fitness landscape versus the optimized target fitness landscape expected from the designed antibody ensemble (the vertical axis from Figure 1E). Not only do we obtain impressive Pearson correlation $r = 0.994$, but fitness is predicted with near exact quantitative accuracy including scaling prefactors, a merit of the biophysical fitness formula eq. (2.7). Combined with our published *in vitro* [10] and genomic [35] evidence to

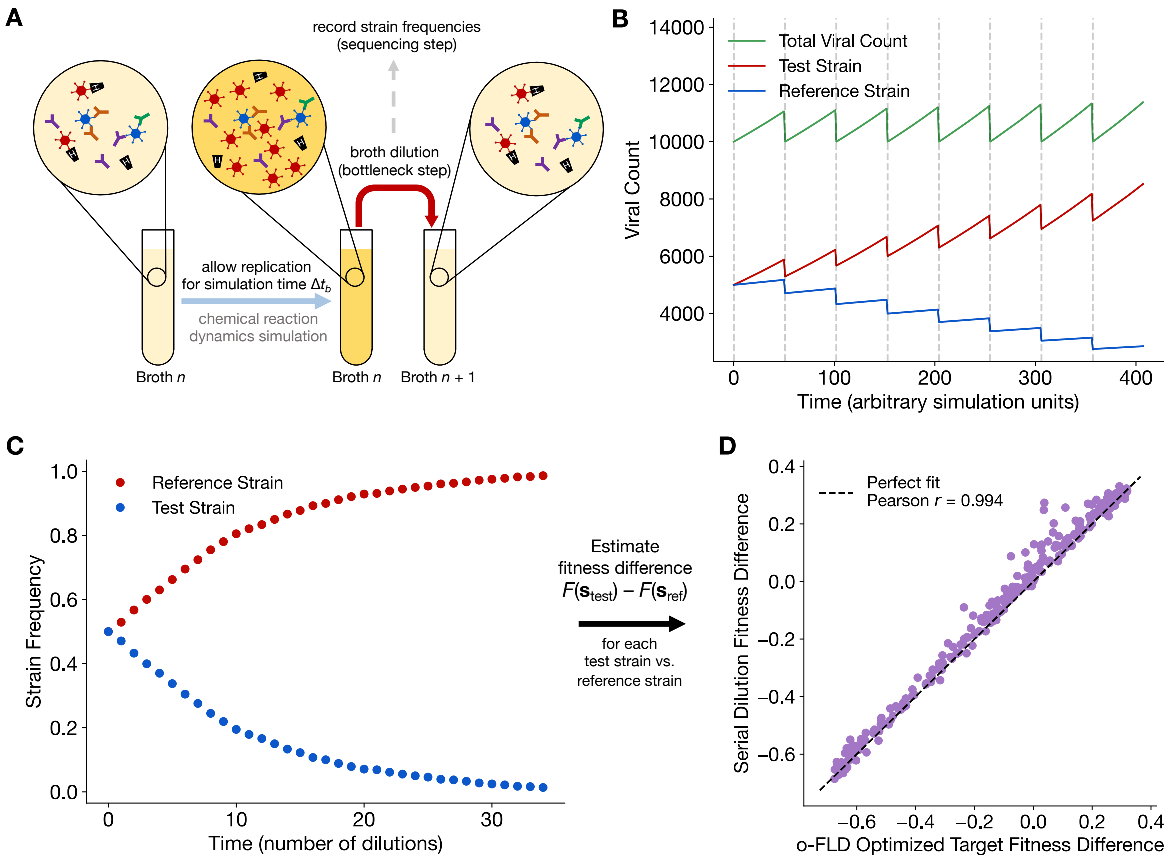

**Figure 2**: ***In silico* serial dilution experiments validate FLD.** (**A**) Schematic of *in silico* serial dilution experiments using microscopic chemical reaction dynamics. (**B**) Time evolution of viral counts from chemical reaction dynamics across multiple broth dilution steps. (**C**) Observable strain frequency data, measured at the broth dilution steps indicated by gray dashed lines in (A) and (B), representing the data available to an experimenter with no knowledge of the underlying microscopic chemical reactions. (**D**) Near-exact agreement between optimized target fitness difference (equivalent to vertical axis from Figure 1E) and simulation fitness difference. Plotted fitnesses are normalized, scaled down by $k_{\mathrm{rep}}(N_o - 1)N_{\mathrm{ent}}$.

support the validity of fitness functions similar to eq. (2.6) (which until now had not been theoretically proven), our *in silico* serial dilution experiments here provide compelling evidence that viruses do evolve according to fitness landscapes designed with eq. (2.7). We now use eq. (2.7) directly for applied design tasks.

### 2.5 Tuning the fitness landscape of two SARS-CoV-2 neutral genotype networks

We now apply the o-FLD protocol to design the fitness landscape of two SARS-CoV-2 neutral genotype networks. The SARS-CoV-2 wildtype strain has 16 possible mutations at residue G485 that result in zero change in fitness. These 17 genotypes (including the wildtype) are all single mutational neighbors of each other, and are referred to as a neutral set, neutral network, or genotype network [36–38]. The Q493R mutation increases fitness of these 17 genotypes

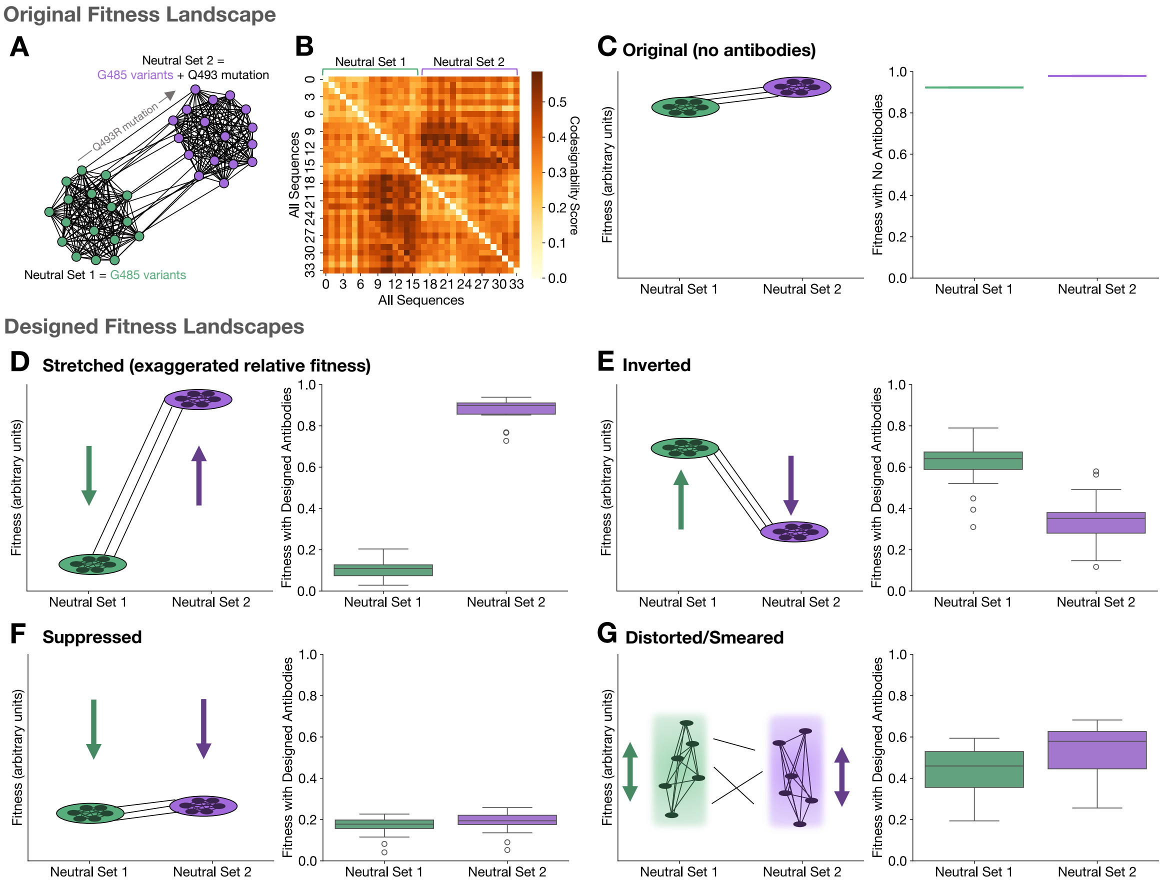

**Figure 3**: **SARS-CoV-2 neutral networks are tunable.** (**A**) Two SARS-CoV-2 neutral networks, each with 17 genotypes variable at residue G485, connected by the fitness-increasing mutation Q493R. (**B**) Codesignability matrix of the 34 genotypes in the 2 neutral networks, showing general trend of codesignability between neutral sets 1 and 2. (**C**) Fitnesses of original neutral networks. (**D**) Stretching relative fitnesses of the neutral sets using o-FLD, making neutral set 2 even more fit relative to neutral set 1. (**E**) Inverting the relative fitnesses of the neutral sets using o-FLD, turning Q493R from a fitness-increasing mutation into a deleterious one. (**F**) Suppressing the fitnesses of both neutral sets using o-FLD, approximately maintaining their relative fitnesses but decreasing absolute fitness. (**G**) Distorting the two neutral networks using o-FLD, breaking the neutrality of the networks altogether. Left-hand panels in (**C**-**F**) show schematics while right-hand panels show box plots of the 34 fitnesses after applying the o-FLD protocol.

without breaking neutrality; it naturally occurred between the wildtype and Omicron variants of SARS-CoV-2 [39]. Thus, the 17 Q493R− and 17 Q493R+ variants at the G485 site form two neutral networks which are interconnected fitness plateaus (Figure 3A).

We computed the codesignability matrix for these 34 genotypes and found that the matrix separates into a $2 \times 2$ block matrix, with the off-diagonal blocks generally darker than the on-diagonal blocks (Figure 3B). This indicates that the two neutral networks, overall, should be codesignable as groups, and sequences within a neutral network are expected to be less codesignable. In Figure 3C, we plot a schematic representation of the two neutral networks as well as their actual fitness distributions, which are flat by definition.

Now, we demonstrate how o-FLD can transform the original fitness landscape into a wide range of user-specified designs. By setting a (scaled) fitness target of 0 for the Q493R− neutral set (neutral set 1) and a target of 1 for the Q493R+ neutral set (neutral set 2), the o-FLD protocol introduced in the previous section dramatically exaggerates the relative fitness differences between the 2 neutral networks, dropping the fitness of neutral set 1 much more than the fitness of neutral set 2 (Figure 3D). By setting a fitness target of 1 for neutral set 1 and a target of 0 for neutral set 2, we can flip the relative fitnesses of the two neutral sets, causing neutral set 2 genotypes to generally have lower fitness than neutral set 1 genotypes (Figure 3E). Q493R can thus be transformed from a fitness-increasing mutation into a deleterious mutation.

By setting fitness targets to 20% of their original values, we suppress the fitnesses of both neutral sets (Figure 3F). Lastly, we cam break the neutrality of both neutral sets by assigning completely random fitnesses chosen from a uniform interval to all 34 genotypes, distorting the fitness landscape entirely (Figure 3G).

FLD thus provides a method for molding fitness landscapes like clay: stretching, inverting, suppressing, and distorting the landscape are made possible through the optimization protocol introduced previously. We emphasize that all of these transformations only ever lower—never increase—fitnesses since the maximum fitness of a strain is determined by its host receptor binding ability, which is not controlled by antibodies. This property aids vaccine design.

#### 2.6 Iterative fitness landscape design protocol discovers better fitness peak-lowering antibodies

We now apply FLD to vaccine design to create evolutionary traps that restrict viral fitness growth. According to eq. (2.7), in the absence of any antibodies, viral fitness can increase by improving host receptor binding, mutating along the fitness landscape toward the fitness peak. After vaccinating against the wildtype or most prevalent strain, this fitness peak can shift (Figure 4A). This immune pressure encourages escape mutations, which decrease antibody-antigen binding affinity.

Given the propensity for escape mutations to occur, we developed a vaccination protocol that plans *ahead*, analogous to a chess player calculating future moves. The goal of our iterative FLD (i-FLD) protocol is to find a vaccine target antigen which optimally suppresses the *post-vaccination* fitness peak (Figure 4B). We first vaccinate against the wildtype/starting strain, obtaining a high-affinity antibody to this strain (the "standard vaccination" protocol). Then we calculate the fitness peak ("Peak #2") of the new fitness landscape in the presence of the wildtype-targeting (WTT) antibody from standard vaccination. The force field calculations are subsequently used to find a high-affinity antibody ("Antibody #2") against Peak #2's antigen sequence. The fitness peak in the presence of Antibody #2 is then targeted for the next iteration.

We continue to iterate this procedure until the new global fitness peak maps back onto a previously discovered one or until a maximum number of iterations is reached. Lastly, the

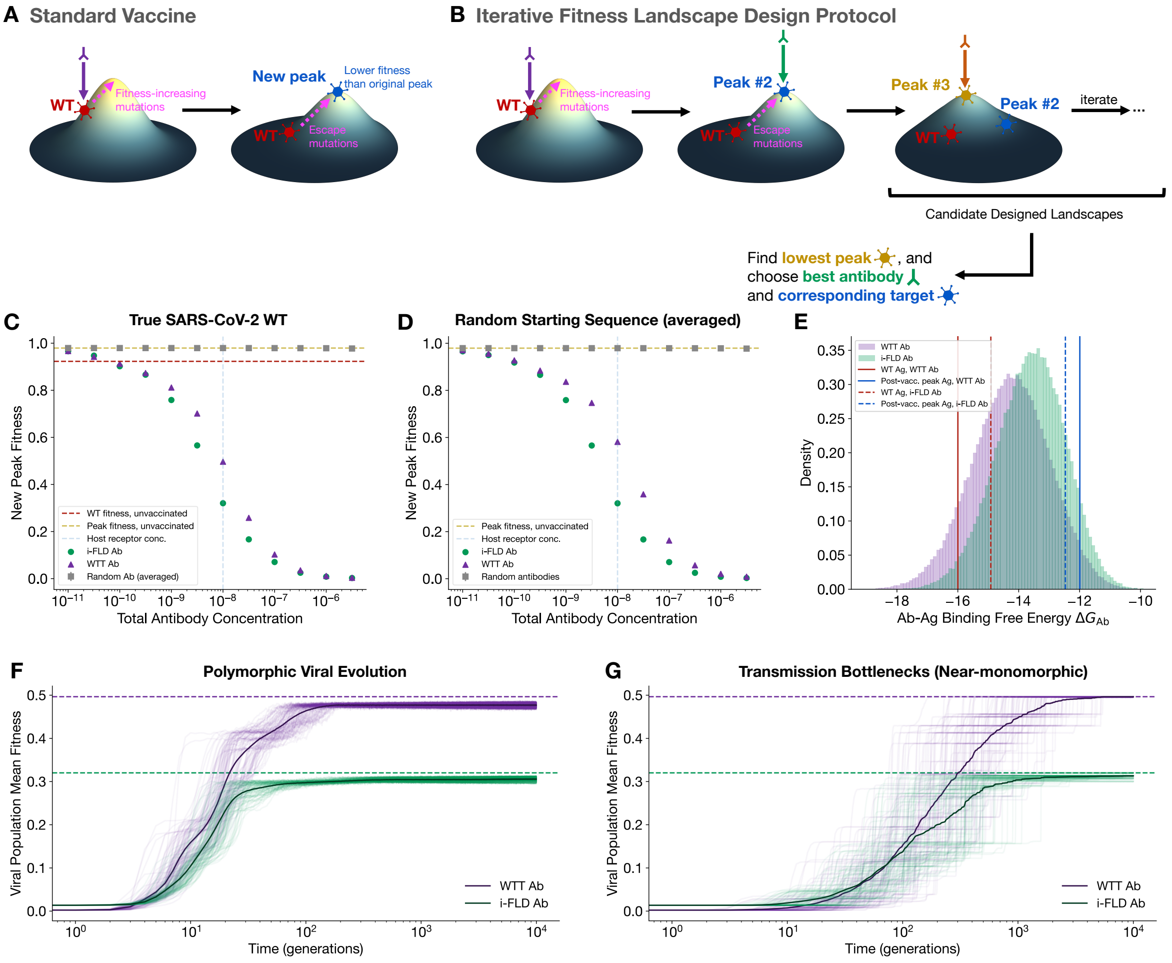

**Figure 4**: **Restricting the peak fitness of viral escape variants.** (**A**) Standard vaccination protocol, generating the wildtype-targeting antibody, targets the wildtype sequence. Viral escape mutations cause fitness increases towards the post-vaccination fitness peak. (**B**) i-FLD finds the vaccine target which minimizes post-vaccination peak fitness through an iterative procedure of targeting the new global fitness peak in the context of the previous antibody. (**C**) Comparison of i-FLD, standard vaccination (WTT antibody), and random antibodies, using the SARS-CoV-2 wildtype as the starting sequence. (**D**) Same as (**C**) but averaged over random starting sequences. (**E**) i-FLD and standard vaccination antibodies' binding affinities to both target antigens. (**F**) Polymorphic and (**G**) near-monomorphic viral evolutionary dynamics demonstrate that populations exhibit slower fitness growth and are trapped under a lower fitness ceiling by the i-FLD antibody compared to the WTT antibody. Dashed lines indicate global maximum fitness, and translucent lines are population mean fitness trajectories from each of 100 Wright-Fisher trials.

protocol identifies the lowest fitness peak, which immediately reveals the ideal antibody and target antigen for fitness peak supression.

To evaluate performance, we calculated the impact of the i-FLD antibody, the WTT antibody, and random antibodies on the post-vaccination global fitness peak, at fixed antibody concentrations (Figure 4C). In general, the i-FLD antibody consistently outperformed the WTT antibody and random antibodies in reducing peak fitness (Figure 4C; extended results in Figure S1A). To see if the same phenomenon would hold if the SARS-CoV-2 wildtype were different (i.e. if the pandemic had begun with a different starting sequence), we repeated the same study for starting sequences chosen at random. Averaging over an approximately uniform distribution of starting fitnesses (see Methods), we found similar results, with the i-FLD antibody reducing the fitness peak better than the WTT antibody and random antibodies (Figure 4D). As expected, these differences in fitness reduction became less prominent in the limits of very high or very low antibody concentration, aside from small fluctations.

To understand why the i-FLD vaccination targets generally work better than the standard vaccination target, we compared the binding free energies of the two antibodies to each protocol's target antigens. Considering the SARS-CoV-2 wildtype case, we found that the i-FLD antibody binds more strongly to the i-FLD target antigen than the WTT antibody does (dashed and solid blue lines in Figure 4E; Figure S1B). The tradeoff is that the i-FLD antibody has to sacrifice some binding affinity to the wildtype strain (dashed and solid red lines in Figure 4E; Figure S1C). Fortunately, this sacrifice is often small enough to ensure that the i-FLD antibody substantially suppresses wildtype fitness, often comparable to the WTT performance (Figure S1D). The i-FLD protocol thus achieves a lower post-vaccination with limited sacrifice of wildtype neutralization.

To ensure that the new vaccination protocol would not inadvertently lead to escape variants with better host binding affinity, we calculated host binding free energies of the post-vaccination global peaks from both protocols. We observed that vaccination by either i-FLD or WTT always forced escape variants to become weaker binders to host receptors compared to the global fitness peak with no antibodies (Figure S1E).

Finally, we simulated viral escape dynamics using the Wright-Fisher model (Methods) in the presence of the WTT antibody or the i-FLD antibody both in the polymorphic regime ($N_{\mathrm{pop}}\mu L_{\mathrm{Ag}} \gg 1$), corresponding to large viral populations $N_{\mathrm{pop}}$ with high mutation rate per site $\mu$ [10], and near the monomorphic regime ($N_{\mathrm{pop}}\mu L_{\mathrm{Ag}} \ll 1$) [9], which we use to represent small infective populations in transmission events. We found that in both polymorphic (Figure 4F and Figure S2A) and near-monomorphic (Figure 4G and Figure S2B) regimes, populations exhibited slower mean fitness growth and were trapped under a lower fitness ceiling by the i-FLD antibody than the WTT antibody.

Together, these results suggest that at the start of the COVID-19 pandemic, vaccinating against the i-FLD-identified target strain would have led to a lower ceiling on the fitness of escape variants, effectively trapping the fitness trajectory of escape variants below a lower fitness threshold. By vaccinating against a future global peak by anticipating escape mutations, the i-FLD protocol generates a tighter-binding antibody to future escape variants with little sacrifice to the neutralization capacity of the wildtype or current dominant variant.

### 3 Discussion

Fitness landscape design represents a major advance toward addressing a fundamental inverse problem in evolutionary biology: the control of evolutionary outcomes, which has wide repercussion for the treatment of any human disease-causing agent which evolves on timescales relevant to human health, including microbial pathogens and cancers. In this work, we showed that a biophysical fitness function for viral surface proteins could be used in a stochastic optimization protocol to design antibody ensembles which achieve user-defined target fitness landscapes. We also introduced an iterative FLD protocol which identifies better vaccine targets that limit future fitness increase of viral escape variants without much sacrifice of wildtype neutralization capacity.

FLD offers a new perspective on vaccine design. Modern rational design of epitope-targeted vaccines focuses substantially on fitness estimation from PCR sequence prevalence data [8, 40, 41]—which would not even exist at the start of a pandemic—on finding high affinity antibodies for specific prevalent antigens [13–15], and on generating broadly neutralizing antibodies through design of antigen cocktails [16, 17]. We show with the i-FLD protocol, however, that even a type of single antibody directed against a prudent choice of target antigen, can impose post-vaccination fitness thresholds on escape variants.

Additionally, this work sets the stage for many directions of applications and advancements stemming from the FLD protocols presented here. In addition to *in vitro* serial dilution experiments, future directions for further developing FLD include using deep learning and large language models for binding affinity prediction [35, 42–44], gradient-based stochastic optimization algorithms enabled by continuous space protein sequence representations [45], small peptide drug design, designing evolutionary fitness traps for cancers through CAR-T cell receptor design, and more. Fitness landscapes are no longer simply visual aids or a theoretical tool, but rather are entities designable using biophysical principles which are capable of shaping longer term evolutionary outcomes.

## 4 Methods

### 4.1 Derivation of the fitness-free energy relation

#### 4.1.1 Binding reactions

We write down the definition of the dissociation constant for eq. (2.2):

$$K_{d,\mathrm{H}}(\mathbf{s}) = \frac{[\mathrm{V}_{\mathrm{ent}}(\mathbf{s})][\mathrm{H}]}{C_0[\mathrm{V}_{\mathrm{ent}}(\mathbf{s})\cdot\mathrm{H}]}, \tag{4.1}$$

and for eq. (2.3)

$$K_{d,\mathrm{Ab}}(\mathbf{s},\mathbf{a}_n) = \frac{[\mathrm{V}_{\mathrm{ent}}(\mathbf{s})][\mathrm{Ab}_n(\mathbf{a}_n)]}{C_0[\mathrm{V}_{\mathrm{ent}}(\mathbf{s})\cdot\mathrm{Ab}_{\mathrm{n}}(\mathbf{a}_{\mathrm{n}})]}., \tag{4.2}$$

where $C_0$ is the reference concentration used to make the dissociation constants dimensionless. We seek an expression for $p_b(\mathbf{s})$, the equilibirum host-bound fraction of viral entry proteins:

$$\begin{aligned} p_b(\mathbf{s}) &\equiv \frac{[\mathrm{V}_{\mathrm{ent}}(\mathbf{s})\cdot\mathrm{H}]}{[\mathrm{V}_{\mathrm{ent}}^{\mathrm{total}}(\mathbf{s})]} \\ &= \frac{[\mathrm{V}_{\mathrm{ent}}(\mathbf{s})\cdot\mathrm{H}]}{[\mathrm{V}_{\mathrm{ent}}(\mathbf{s})]+[\mathrm{V}_{\mathrm{ent}}(\mathbf{s})\cdot\mathrm{H}]+[\mathrm{V}_{\mathrm{ent}}(\mathbf{s})\cdot\mathrm{Ab}_{\mathrm{n}}(\mathbf{a}_{\mathrm{n}})]}. \end{aligned} \tag{4.3}$$

Substituting using eq. (4.1) and eq. (4.2), we obtain:

$$p_b(\mathbf{s}) = \frac{\frac{[\mathrm{V_{ent}}(\mathbf{s})][\mathrm{H}]}{C_0 K_{d,\mathrm{H}}(\mathbf{s})}}{[\mathrm{V_{ent}}(\mathbf{s})] + \frac{[\mathrm{V_{ent}}(\mathbf{s})][\mathrm{H}]}{C_0 K_{d,\mathrm{H}}(\mathbf{s})} + \frac{[\mathrm{V_{ent}}(\mathbf{s})][\mathrm{Ab}_n(\mathbf{a}_n)]}{C_0 K_{d,\mathrm{Ab}}(\mathbf{s},\mathbf{a}_n)}} = \frac{\frac{[\mathrm{H}]}{K_{d,\mathrm{H}}(\mathbf{s})}}{C_0 + \frac{[\mathrm{H}]}{K_{d,\mathrm{H}}(\mathbf{s})} + \frac{[\mathrm{Ab}_n(\mathbf{a}_n)]}{K_{d,\mathrm{Ab}}(\mathbf{s},\mathbf{a}_n)}} \tag{4.4}$$

Using $\Delta G_\mathrm{H}(\mathbf{s}) = \beta^{-1} \log K_{d,\mathrm{H}}(\mathbf{s})$ as well as $\Delta G_\mathrm{Ab}(\mathbf{s}, \mathbf{a}_n) = \beta^{-1} \log K_{d,\mathrm{Ab}}(\mathbf{s}, \mathbf{a}_n)$, we arrive at

$$p_b(\mathbf{s}) = \frac{[\mathrm{H}] e^{-\beta \Delta G_\mathrm{Ab}(\mathbf{s})}}{C_0 + [\mathrm{H}] e^{-\beta \Delta G_\mathrm{H}(\mathbf{s})} + \sum_n [\mathrm{Ab}_n(\mathbf{a}_n)] e^{-\beta \Delta G_\mathrm{Ab}(\mathbf{s},\mathbf{a}_n)}}. \tag{4.5}$$

$[\mathrm{H}]$ and $[\mathrm{Ab}_n(\mathbf{a}_n)]$ are unbound host and antibody concentrations.

We assume that the availability of infectable host cells and receptors is much higher than the viral load, and clinical data suggests that antibody concentrations are much higher than the viral concentration in a immunocompetent, vaccinated individual (Supplementary Note 1). Assuming that host receptors and antibodies are not significantly depleted by viral binding, we can approximately equate the free and experimentally controllable total concentrations $[\mathrm{H}^\mathrm{total}] \approx [\mathrm{H}]$ and $[\mathrm{Ab}_n^\mathrm{total}(\mathbf{a}_n)] \approx [\mathrm{Ab}_n(\mathbf{a}_n)]$. Thus, we have

$$p_b(\mathbf{s}) \approx \frac{[\mathrm{H}^\mathrm{total}] e^{-\beta \Delta G_\mathrm{Ab}(\mathbf{s})}}{C_0 + [\mathrm{H}^\mathrm{total}] e^{-\beta \Delta G_\mathrm{H}(\mathbf{s})} + \sum_n [\mathrm{Ab}_n^\mathrm{total}(\mathbf{a}_n)] e^{-\beta \Delta G_\mathrm{Ab}(\mathbf{s},\mathbf{a}_n)}}, \tag{4.6}$$

which is eq. (2.6).

#### 4.1.2 Replication dynamics

We note that the chemical reaction eq. (4.7) produces *unbound* copies of the virus, which we temporarily denote as $V(\mathbf{s}) \cdot (0\,\mathrm{H})$ for mathematical convenience. Thus, we have

$$\mathrm{V}(\mathbf{s}) \cdot (j\,\mathrm{H}) \xrightarrow{j\,k_\mathrm{rep}} N_o\,\mathrm{V}(\mathbf{s}) \cdot (0\,\mathrm{H}). \tag{4.7}$$

The ordinary differential equation for the time evolution of the unbound concentration must include contributions from the rate equations for values of all $j \in \{1, \ldots, N_\mathrm{ent}\}$:

$$\frac{\mathrm{d}[\mathrm{V}(\mathbf{s}) \cdot (0\ \mathrm{H})]}{\mathrm{d}t} = N_o \sum_{j=1}^{N_\mathrm{ent}} (j k_\mathrm{rep}) [\mathrm{V}(\mathbf{s}) \cdot (j\ \mathrm{H})]. \tag{4.8}$$

The same reaction equation also provides the depletion rate of the replicating virus bound to $j$ host receptors:

$$\frac{\mathrm{d}[\mathrm{V}(\mathbf{s}) \cdot (j\ \mathrm{H})]}{\mathrm{d}t} = -(j k_\mathrm{rep}) [\mathrm{V}(\mathbf{s}) \cdot (j\ \mathrm{H})], \quad 1 \le j \le N_\mathrm{ent} \tag{4.9}$$

We now denote the total viral concentration for strain $\mathbf{s}$ as

$$[\mathrm{V}^{\mathrm{tot}}(\mathbf{s})] = \sum_{j=0}^{N_{\mathrm{ent}}} [\mathrm{V}(\mathbf{s}) \cdot (j\ \mathrm{H})], \tag{4.10}$$

which includes all viruses in all bound and unbound states. Taking a time derivative, it immediately follows that

$$\begin{aligned} \frac{\mathrm{d}[\mathrm{V}^{\mathrm{tot}}(\mathbf{s})]}{\mathrm{d}t} &= \sum_{j=0}^{N_{\mathrm{ent}}} \frac{\mathrm{d}[\mathrm{V}(\mathbf{s}) \cdot (j\ \mathrm{H})]}{\mathrm{d}t} \\ &= \frac{\mathrm{d}[\mathrm{V}(\mathbf{s}) \cdot (0\ \mathrm{H})]}{\mathrm{d}t} + \sum_{j=1}^{N_{\mathrm{ent}}} \frac{\mathrm{d}[\mathrm{V}(\mathbf{s}) \cdot (j\ \mathrm{H})]}{\mathrm{d}t}. \end{aligned} \tag{4.11}$$

Plugging in the results of eq. (4.8) and eq. (4.9), we immediately have

$$\begin{aligned} \frac{\mathrm{d}[\mathrm{V}^{\mathrm{tot}}(\mathbf{s})]}{\mathrm{d}t} &= N_o \sum_{j=1}^{N_{\mathrm{ent}}} (j k_{\mathrm{rep}}) [\mathrm{V}(\mathbf{s}) \cdot (j\ \mathrm{H})] - \sum_{j=1}^{N_{\mathrm{ent}}} (j k_{\mathrm{rep}}) [\mathrm{V}(\mathbf{s}) \cdot (j\ \mathrm{H})] \\ &= (N_o - 1) k_{\mathrm{rep}} \sum_{j=1}^{N_{\mathrm{ent}}} j [\mathrm{V}(\mathbf{s}) \cdot (j\ \mathrm{H})]. \end{aligned} \tag{4.12}$$

We now invoke separation of timescales: if the binding dynamics occur at timescales much faster than replication, then $[\mathrm{V}(\mathbf{s}) \cdot (j\ \mathrm{H})]$ can be calculated at equilibrium. At equilibrium, we showed that the probability of finding any viral entry protein bound to a host receptor is $p_b(\mathbf{s})$. Thus, the number of entry proteins on a single virion which are bound to host receptors is a binomial random variable $J \sim \mathrm{Binomial}(N_{\mathrm{ent}}, p_b(\mathbf{s}))$. The fraction of virions bound to $j$ hosts should follows the distribution of $j$:

$$[\mathrm{V}(\mathbf{s}) \cdot (j\ \mathrm{H})] = [\mathrm{V}^{\mathrm{tot}}(\mathbf{s})] \mathbb{P}[J = j] \tag{4.13}$$

Substituting into eq. (4.12), it follows that

$$\begin{aligned} \frac{\mathrm{d}[\mathrm{V}^{\mathrm{tot}}(\mathbf{s})]}{\mathrm{d}t} &= (N_o - 1) k_{\mathrm{rep}} [\mathrm{V}^{\mathrm{tot}}(\mathbf{s})] \sum_{j=1}^{N_{\mathrm{ent}}} j \mathbb{P}[J = j] \\ &= (N_o - 1) k_{\mathrm{rep}} [\mathrm{V}^{\mathrm{tot}}(\mathbf{s})] \sum_{j=0}^{N_{\mathrm{ent}}} j \mathbb{P}[J = j]. \end{aligned} \tag{4.14}$$

Noting that the sum is exactly the expectation of a binomial variable, we have

$$\sum_{j=0}^{N_{\mathrm{ent}}} j \mathbb{P}[J = j] = \mathbb{E}[J] = N_{\mathrm{ent}} p_b(\mathbf{s}), \tag{4.15}$$

which provides the final result

$$\frac{\mathrm{d}[\mathrm{V}^{\mathrm{tot}}(\mathbf{s})]}{\mathrm{d}t} = (N_o - 1) k_{\mathrm{rep}} N_{\mathrm{ent}} p_b(\mathbf{s}) [\mathrm{V}^{\mathrm{tot}}(\mathbf{s})], \tag{4.16}$$

which is eq. (2.5), the desired result.

#### 4.2 Fitness calculations

*Force field binding free energy calculations.* We obtained PDB structures for the antigen-antibody complex (PDB number 7KMG) and antigen-host complex (PDB number 7DQA). 4 antigen residues, 5 antibody light chain residues, and 6 antibody heavy chain residues (Table S1) were chosen to be the mutable residues for fitness landscape design. Each selected antigen residue was involved in contact with both the antibody and the host, defined by $< 8$ Å distance between $C_\alpha$ atoms. We exhaustively computed host-antigen binding free energies $\Delta G_{\mathrm{H}}(\mathbf{s})$ for all $20^4 = 160,000$ antigenic variants using EvoEF force field [27] calculations performed at default (room) temperature, consistent with calculation of $\beta$ in eq. (2.7) and BioNetGen simulations.

*Potts model for antibody-antigen binding free energy.* For the antigen-antibody complex, mutations could occur at the 4 antigenic sites and at all 11 antibody sites, so with $20^{4+11} \approx 3.28 \times 10^{19}$ possible variants, exhaustive free energy calculation is impossible. So, we randomly sampled 160,000 variants and computed $\Delta G_{\mathrm{Ab}}(\mathbf{s}, \mathbf{a})$ using EvoEF. We used these data to train the parameters of a site-symmetric, spin-asymmetric chiral Potts model:

$$\Delta G_{\mathrm{Ab}}^{\mathrm{Potts}}(\boldsymbol{\sigma}) = G_0 + \sum_{i=1}^{4} h_i(\sigma_i) + \sum_{i=1}^{4} \sum_{j=1}^{4} \left[ J_{ij}(\sigma_i, \sigma_j) + J_{ji}(\sigma_i, \sigma_j) \right], \tag{4.17}$$

where $\boldsymbol{\sigma}$ is a length 15 vector combining antigen sequence $\mathbf{s}$ and antibody sequence $\mathbf{a}$. Using a 80%-20% training-validation split, we used stochastic gradient descent to train the 90,301 parameters $G_0$, $\{h_i(\sigma_i)\}$, and $\{J_{ij}(\sigma_i, \sigma_j)\}$. The Potts model achieved internal consistency, with training Pearson $r = 0.935$ (Figure S4A) and validation $r = 0.914$ (Figure S4B).

*Calibrating free energy predictions to experimental data.* Ref. [28] conducted a deep mutational scan across the SARS-CoV-2 spike RBD and reported binding free energies. Since their mutations were not fully combinatorial, we extracted the 77 sequences in their dataset which overlapped with our antigenic sequences (19 single-site mutations at each of the 4 antigenic sites, plus the wildtype). Experimental $\Delta G_{\mathrm{H}}(\mathbf{s})$ measurements are plotted against the force field calculations in Figure S3A. We calibrated the force field calculations to experimental measurements by moment-matching. By computing the means $\mu_{\Delta G}$ and standard deviations $\sigma_{\Delta G}$ of both force field and experimental overlapping datasets, we applied the linear transformation

$$\Delta \widetilde{G} = \left[ \mu_{\Delta G_{\text{experiment}}} - \mu_{\Delta G_{\text{force field}}} \left( \frac{\sigma_{\Delta G_{\text{experiment}}}}{\sigma_{\Delta G_{\text{force field}}}} \right) \right] + \frac{\sigma_{\Delta G_{\text{experiment}}}}{\sigma_{\Delta G_{\text{force field}}}} \Delta G_{\text{force field}} \tag{4.18}$$

to obtain the calibrated $\Delta \widetilde{G}_{\mathrm{H}}(\mathbf{s})$ force field binding free energies (Figure S3B). This calibration allows the means and standard deviations of the overlapping data distribution to match between force field and experiment (Figure S3C). The same calibration equation was then applied to the antibody-antigen Potts model (Figure S4C and D).

*Fitness from free energy calculations.* Calibrated $\Delta \widetilde{G}_{\mathrm{H}}(\mathbf{s})$ and calibrated Potts model-predicted $\Delta \widetilde{G}_{\mathrm{Ab}}^{\mathrm{Potts}}(\mathbf{s}, \mathbf{a})$ were plugged into eq. (2.7) to compute fitness throughout this work.

#### 4.3 Computing fitness landscape designability phase diagram and codesignability matrix

To compute a fitness landscape designability phase diagram and codesignability matrix, we randomly sampled the space of antibody ensembles. Antibodies were initialized with random paratope sequences at the 11 mutable sites, and starting concentrations were initialized over a broad range of order of magnitudes, heuristically adjusting the range to ensure that all fitnesses were not all close to 0 or all close to maximal. We sampled 10,000 sets of random antibody ensembles to produce the results in Figure 1A and B, where we used 3 antibodies, and for Figure 3B, where we used 4 antibodies. For any sequence pair (or triplet), we used a support vector machine (SVM) with a radial basis function kernel to identify a decision boundary separating designable and undesignable regions. This was implemented with Scikit-learn's [46] OneClassSVM, with parameters $\gamma = 5$ ($\gamma = 6$ for the 3D designability diagrams) and $\nu = 0.0001$. Codesignability scores for sequence pairs were calculated by splitting the unit square into a $100 \times 100$ grid on which the SVM decision was evaluated, and then summing the area of the squares where the decision was

#### 4.4 Stochastic optimization for fitness landscape design (o-FLD protocol)

The o-FLD protocol uses a mean-square-error (MSE) loss (multiplied by a factor of $10,000$ for numerical convenience) between the biophysical fitness landscape $F_{\text{target}}(\mathbf{s})$ from eq. (2.7) and a target fitness landscape $F_{\text{target}}(\mathbf{s})$. We initialized random antibody ensembles by picking random amino acids at the mutable positions. We then picked random starting concentrations which were some random number between 0 and 1 multiplied by $10^{\eta^*}$, where $\eta^*$ is the exponent which minimized the loss for that starting set of random residues; for Figure 3E, we instead initialized with a remapped $\eta^* \mapsto \eta^* + 1$ because this tended to improve convergence to a deeper minimum.

We then perform simulated annealing, using the Metropolis-Hastings algorithm [32], with an exponential cooling schedule starting with simulation temperature $T_{\text{MCMC}} = 10$ and dividing by a factor of 10 after every 10,000 time steps until the 10,000 steps at the final simulation temperature (0.1 or 0.01) were completed. At each time step, we first proposed the swap of a random mutable residue of a random antibody to a random amino acid. The scaled MSE loss was recalculated, and the proposal was accepted or rejected with the standard Metropolis acceptance probability

$$\begin{aligned} &A(\{\mathbf{a}_n\}, \{[\text{Ab}_n(\mathbf{a}_n)]\} \to \{\mathbf{a}_n^{\text{update}}\}, \{[\text{Ab}_n(\mathbf{a}_n)]\}) \\ &= \min\left(1, e^{-\frac{10,000}{T_{\text{sim}}}\left(\mathcal{L}_{\text{MSE}}(\{\mathbf{a}_n^{\text{update}}\},\{[\text{Ab}_n(\mathbf{a}_n)]\})-\mathcal{L}_{\text{MSE}}(\{\mathbf{a}_n\},\{[\text{Ab}_n(\mathbf{a}_n)]\})\right)}\right) \end{aligned} \tag{4.19}$$

We then proposed a concentration update by randomly increasing or decreasing each concentration by $10^{\eta^*-1}C_0$ if the simulation temperature was $T_{\text{MCMC}} \in \{10, 1, 0.1\}$ or by $10^{\eta^*-2}C_0$

if $T_{\text{MCMC}} = 0.01$. The concentration update was accepted with probabiltiy

$$A(\{\mathbf{a}_n\}, \{[\text{Ab}_n(\mathbf{a}_n)]\} \to \{\mathbf{a}_n\}, \{[\text{Ab}_n^{\text{update}}(\mathbf{a}_n)]\}) \\ = \min\left(1, e^{-\frac{10{,}000}{T_{\text{sim}}}\left(\mathcal{L}_{\text{MSE}}(\{\mathbf{a}_n\},\{[\text{Ab}_n^{\text{update}}(\mathbf{a}_n)]\}) - \mathcal{L}_{\text{MSE}}(\{\mathbf{a}_n\},\{[\text{Ab}_n(\mathbf{a}_n)]\})\right)}\right). \tag{4.20}$$

The o-FLD algorithm thus uses alternating antibody sequence and concentration updates to find an ideal ensemble for matching the target fitness landscape.

### 4.5 *In silico* serial dilution experiments using chemical reaction dynamics

We used the "bionetgen" Python package to generate BioNetGen [33] simulation files for each serial dilution experiment. For BioNetGen's ODE simulator, concentrations use arbitrary units, as do replication rate constants; the time scale of simulations is set by the rate constants. The host concentration (or count, as the distinction is arbitrary here) was initialized at $10^9$, antibody concentrations were proportional to the ones found using the o-FLD protocol and scaled appropriately depending on the host concentration, $N_o$ was chosen to be 5, $N_{\text{ent}}$ was set to 2 for computational tractability, $k_{\text{rep}}$ was chosen to be $10^{-11}$, and the simulation time within each broth was $\Delta t_b = 3 \times 10^9$. Binding dynamics needed to be faster than replication dynamics; since only dissociation constants $K_d = k_{\text{off}}/k_{\text{on}}$ were prescribed by the force field calculations, we chose $k_{\text{on}} = 10^6$ with $k_{\text{off}} = K_d \times k_{\text{on}}$. Our chosen $k_{\text{rep}}$ was appropriately much smaller than the binding rate constants.

For the first broth, viral concentrations were each initialized at 5,000. After running the BioNetGen ODE simulator for $\Delta t_b$, we computed viral concentrations and rounded them to the nearest integer (to approximately discretize non-integer concentrations to a viral count). We then sampled 10,000 virions, without replacement, to initialize the next broth. These viral counts, divided by the total count of 10,000, were recorded in the strain frequency timeseries. A maximum number of 50 broths were simulated for each test sequence, with broth transfers ending early if the either of the viral counts dropped below 100.

Given test strain frequency $p(t)$ as a function of broth dilution index $t$ and a total number of dilutions $t_{\max}$, the fitness difference $F(\mathbf{s}_{\text{test}}) - F(\mathbf{s}_{\text{reference}})$ was estimated from the strain frequency timeseries with

$$F(\mathbf{s}_{\text{test}}) - F(\mathbf{s}_{\text{reference}}) \approx \frac{1}{t_{\max}\Delta t_b} \frac{p(t_{\max}) - p(0)}{\langle p(t)\rangle - \langle p(t)^2\rangle}, \tag{4.21}$$

where $\langle \cdot \rangle$ is a time average. This is derived in Supplementary Note 2 from the 1-dimensional Kimura equation [6], which is the diffusion limit of population genetics.

### 4.6 Iterative fitness landscape design (i-FLD) protocol

The i-FLD protocol first finds an antibody which binds with high affinity to the starting strain (the WTT antibody). This is accomplished by using simulated annealing as used in the o-FLD protocol, but with the following main modifications: (1) only a single antibody is used, (2) the antibody is at fixed concentration, so the concentration update steps are irrelevant, and (3) the loss function is set to the binding free energy $\Delta\widetilde{G}_{\text{Ab}}^{\text{Potts}}(\mathbf{s},\mathbf{a})$. The simulation temperatures are $T_{MCMC} = 0.1$ followed by 0.01. The simulated annealing generates the WTT antibody, which we then use to calculate the full fitness landscape over all antigenic sequences. We identify the peak fitness and its antigenic sequence, which is set as the target sequence, and we once again run simulated annealing to find a high affinity antibody. A total of 5 iterations after finding the WTT antibody are performed.

For the cases where we used random starting sequences instead of the true SARS-CoV-2 wildtype, we performed a total of 200 random trials. 20 trials had a starting random (normalized) fitness between 0 and 0.1, 20 trials had starting fitness bewteen 0.1 and 0.2, and so forth. We averaged over all 200 trials to produce Figure 4D.

*Wright-Fisher viral evolutionary dynamics.* Wright-Fisher simulations follow the protocol in ref. [9], where we initialize $N_{\text{pop}} = 1000$ antigen sequences at the SARS-CoV-2 wildtype. At each of the 10,000 time steps, we calculate all fitnesses for every individual in the population using eq. (2.7). The next generation of 10,000 individuals is selected by sampling the previous generation's individuals with replacement, with probability $p_i = F_i / \sum_{j=1}^{10{,}000} F_j$, where $i$ indexes the previous generation's individuals. Each of the new generation's sequence sites are chosen for possible mutation with probability $\mu = 0.01$ (for the polymorphic simulations) or $\mu_{\text{chosen}} = 0.0001$ (for the near-monomorphic simulations). If a site is chosen to mutate, a random amino acid is chosen for that site. This makes the mutation probability $\mu = \mu_{\text{chosen}} \times 19/20$. The next time step then begins with fitness calculations of all sequences, which are stored. Since for each antigen we consider $L_{\text{Ag}} = 4$ residues, the polymorphic dynamics have $N_{\text{pop}}\mu L_{\text{Ag}} = 38$, and the near-monomorphic dynamics have $N_{\text{pop}}\mu L_{\text{Ag}} = 0.38$.

### Acknowledgements

We acknowledge helpful discussions with Anna Sappington, Dianzhuo Wang, Aniruddha Chattaraj, Alexander Kelser, and Bidyut K. Mohanty.

*Funding and resource acknowledgement.* This work was supported by awards T32GM007753 and T32GM144273 from the National Institute of General Medical Sciences, a Hertz Foundation Fellowship (VM), and a PD Soros Fellowship (VM). The content is solely the responsibility of the authors and does not necessarily represent the official views of the National Institute of General Medical Sciences or the National Institutes of Health. We thank the MIT Engaging Cluster, supported by IBM, and the Harvard Faculty of Arts and Science Research Computing (FASRC) for computational resources. The authors declare no known conflict of interest.

*Code and data availability.* Code and data will be made publicly available upon publication.

## Supplementary Information

### Supplementary Table

| Molecule | Mutable Residues |
|---|---|
| SARS-CoV-2 Spike Protein | E484, G485, F486, Q493 |
| Ly-CoV555 Light Chain | Y32, S91, Y92, S93, T94 |
| Ly-CoV555 Heavy Chain | Y101, E102, A103, R104, Y109, Y110 |

**Table S1**: Mutable antigen and antibody residues used for fitness landscape design.

### Supplementary Notes

***Supplementary Note 1:*** *Justficiation of antibody and viral receptor relative concentrations from clinical data.* We note that a 50-year-old's mean peak viral load from a SARS-CoV-2 infection—independent of vaccination status—is $10^{8.14} \approx 1.28 \times 10^8$ copies/mL [47]. Each SARS-CoV-2 virion has, on average, $24 \pm 9$ spike protein trimers, so there are on average $72 = 7.2 \times 10^1$ spike proteins per virion [48]. Together, this suggests that an infected individual has $[\mathrm{V_{ent}}(\mathbf{s})] \approx (1.28 \times 10^8) \times (7.2 \times 10^1) \approx 9.22 \times 10^9$ proteins/mL.

Ref. [49] found that previously uninfected individuals who received 2 doses of the SARS-CoV-2 mRNA vaccine had median spike protein-specific IgG concentration of 46.80 $\mu$g/mL, with previously infected but unvaccinated invidiuals having roughly 1 order of magnitude lower concentration and vaccinated individuals who had also been previously infected having roughly 1 order of magnitude greater concentration. Since IgGs have an average molecular weight of 150 kDa (kg/mol) [50], we approximately have

$$\frac{46.8\ \mu\mathrm{g}}{1\ \mathrm{mL}} \times \frac{1\ \mathrm{mol}}{150 \times 10^9\ \mu\mathrm{g}} \times \frac{6.02 \times 10^{23}\ \mathrm{antibodies}}{1\ \mathrm{mol}} = 1.88 \times 10^{14}\ \mathrm{antibodies/mL}, \qquad (4.22)$$

suggesting that antibody concentration outweighs viral concentration. Even with up to $10^2$-$10^3$ orders of magnitude of epitope diversity, the estimated antibody concentration outweighs SARS-CoV-2 spike proteins.

The total host concentration is the sum of bound and unbound host concentrations $[\mathrm{H^{total}}] = [\mathrm{H}] + [\mathrm{V_{ent}}(\mathbf{s}) \cdot \mathrm{H}]$, and the total antibody concentration is the sume of bound and unbound host concentrations $[\mathrm{Ab}_n^{\mathrm{total}}(\mathbf{a}_n)] = [\mathrm{Ab}_n(\mathbf{a}_n)] + [\mathrm{V_{ent}}(\mathbf{s}) \cdot \mathrm{Ab_n}(\mathbf{a}_\mathrm{n})]$. Since we estimate antibody concentrations are greater than the number of viral proteins by orders of magnitude and assume vast availability of infectable host cells, we can write $[\mathrm{V_{ent}}(\mathbf{s}) \cdot \mathrm{H}] < [\mathrm{V_{ent}^{total}}(\mathbf{s})] \ll [\mathrm{H}]$ and $[\mathrm{V_{ent}}(\mathbf{s}) \cdot \mathrm{Ab_n}(\mathbf{a}_\mathrm{n})] < [\mathrm{V_{ent}^{total}}(\mathbf{s})] \ll [\mathrm{Ab}_n^{\mathrm{total}}(\mathbf{a}_n)]$. Therefore, we can approximately equate the free and experimentally controllable total concentrations $[\mathrm{H^{total}}] \approx [\mathrm{H}]$ and $[\mathrm{Ab}_n^{\mathrm{total}}(\mathbf{a}_n)] \approx [\mathrm{Ab}_n(\mathbf{a}_n)]$.

***Supplementary Note 2:*** *Fitness estimation from strain frequency time series.* When there are two strains (test and reference), and $p(t)$ is the test strain frequency as a function of time

$t$, the mean rate of change of the strain frequency—in continuous time—is given by the 1-dimensional Kimura equation [6] in the limit of no stochastic fluctuations (equivalently, the infinite population limit):

$$\frac{\partial p(t)}{\partial t} = p(t)(1 - p(t))(F(\mathbf{s}_{\text{test}}) - F(\mathbf{s}_{\text{reference}})). \tag{4.23}$$

First, we account for the fact that the *in silico* serial dilution experiment timescale is different from the broth dilution time scale. Any exponential growth during a time $\Delta t_b$ in the simulation time looks like unit time in the broth time series. Thus, the fitnesses in the equation above need to be multiplied by a factor of $\Delta t_b$. Thus, we have

$$\frac{\partial p(t)}{\partial t} = \Delta t_b p(t)(1 - p(t))F(\mathbf{s}_{\text{test}}) - F(\mathbf{s}_{\text{reference}}). \tag{4.24}$$

Now, time averaging over both sides of the equation from $[0, t_{\text{max}}]$, we have

$$\begin{aligned} \frac{1}{t_{\text{max}}} \int_0^{t_{\text{max}}} \mathrm{d}t \, \frac{\partial p(t)}{\partial t} &= \frac{\Delta t_b}{t_{\text{max}}} \int_0^{t_{\text{max}}} \mathrm{d}t \, p(t)(1 - p(t)) \left(F(\mathbf{s}_{\text{test}}) - F(\mathbf{s}_{\text{reference}})\right) \\ \Rightarrow \frac{p(t_{\text{max}}) - p(0)}{t_{\text{max}}} &= \Delta t_b \left( \langle p(t) \rangle - \langle p(t)^2 \rangle \right) \left(F(\mathbf{s}_{\text{test}}) - F(\mathbf{s}_{\text{reference}})\right), \end{aligned} \tag{4.25}$$

which is rearranged to

$$F(\mathbf{s}_{\text{test}}) - F(\mathbf{s}_{\text{reference}}) \approx \frac{1}{t_{\text{max}} \Delta t_b} \frac{p(t_{\text{max}}) - p(0)}{\langle p(t) \rangle - \langle p(t)^2 \rangle}. \tag{4.26}$$

The same formula derived with forward-time finite difference will give time averages which are off by a boundary term in the series. We have not investigated the effect of deriving the above formula with an integral versus a sum but do not expect fitness estimation to be substantially affected.

### Supplementary Figures

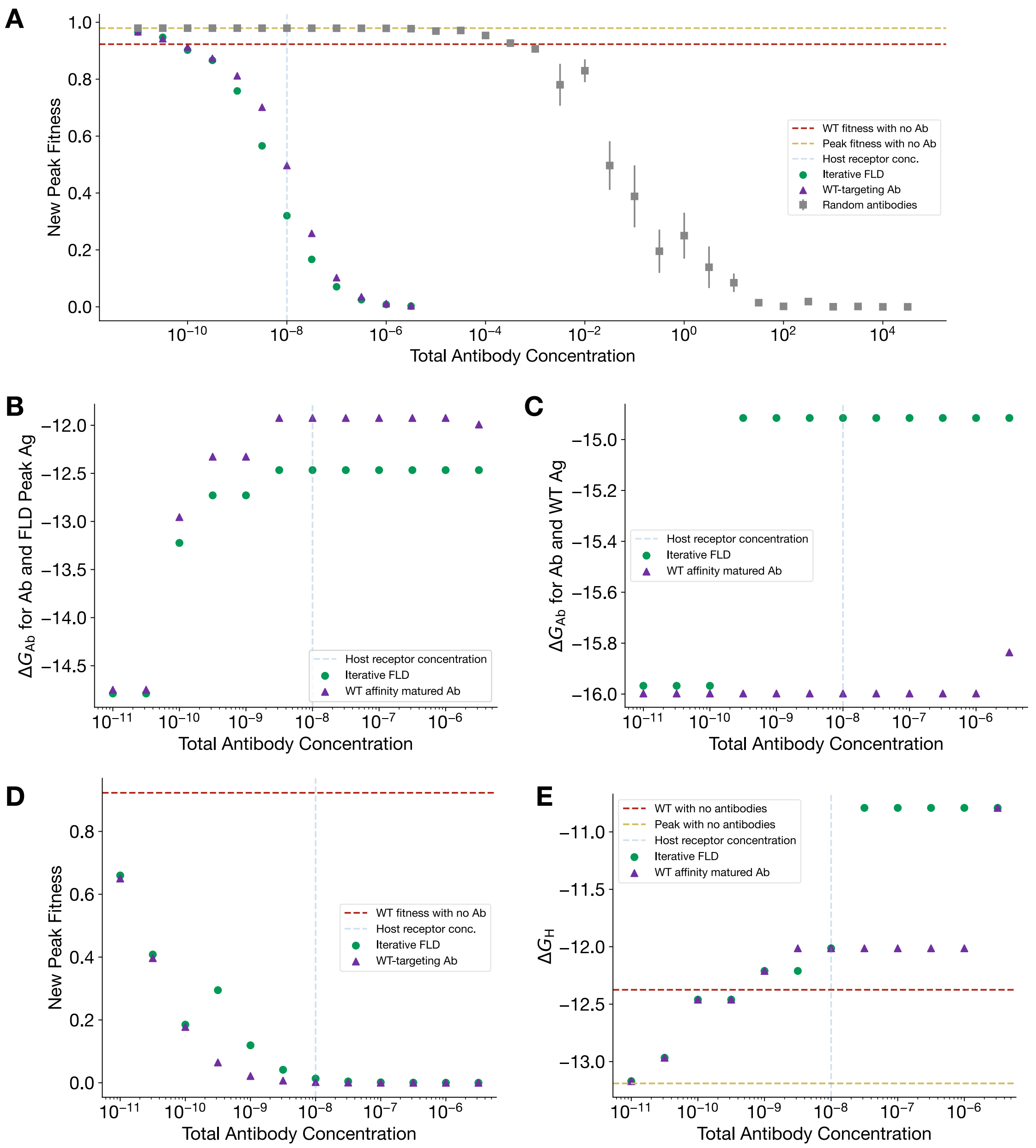

**Figure S1**: **Extended data for i-FLD protocol with SARS-CoV-2 wildtype.** (**A**) Extended range of random antibody concentrations show fitness peak reduction is only possible at concentrations much higher than host concentration. (**B**) The i-FLD antibody binds tighter to its target antigen than the WTT antibody does. (**C**) The i-FLD antibody binds less strongly to the wildtype antigen than the WTT antibody does. (**D**) Despite weaker binding to the wildtype antigen, the i-FLD still substantially suppresses wildtype fitness, often reducing it as much as the WTT antigen. (**E**) Both the i-FLD antibody and the WTT antibody result in post-vaccination fitness peaks for which the new peak antigens bind the host receptor less strongly than in absence of any antibody.

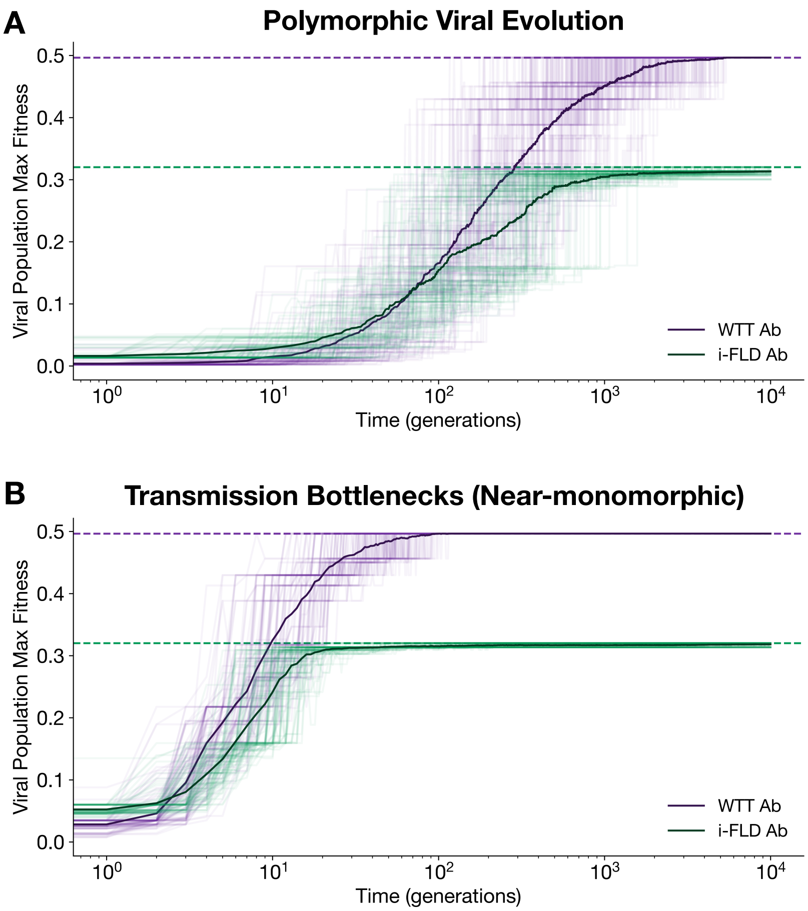

**Figure S2**: **Trajectories of population maximum fitness.** (**A**) Polymorphic and (**B**) near-monomorphic viral evolutionary dynamics, with trajectories corresponding to the highest fitness in the population at any given time. In both regimes, populations exhibit slower fitness growth and are trapped under a lower fitness ceiling by the i-FLD antibody compared to the WTT antibody. Dashed lines indicate global maximum fitness, and translucent lines are population mean fitness trajectories from each of 100 Wright-Fisher trials.

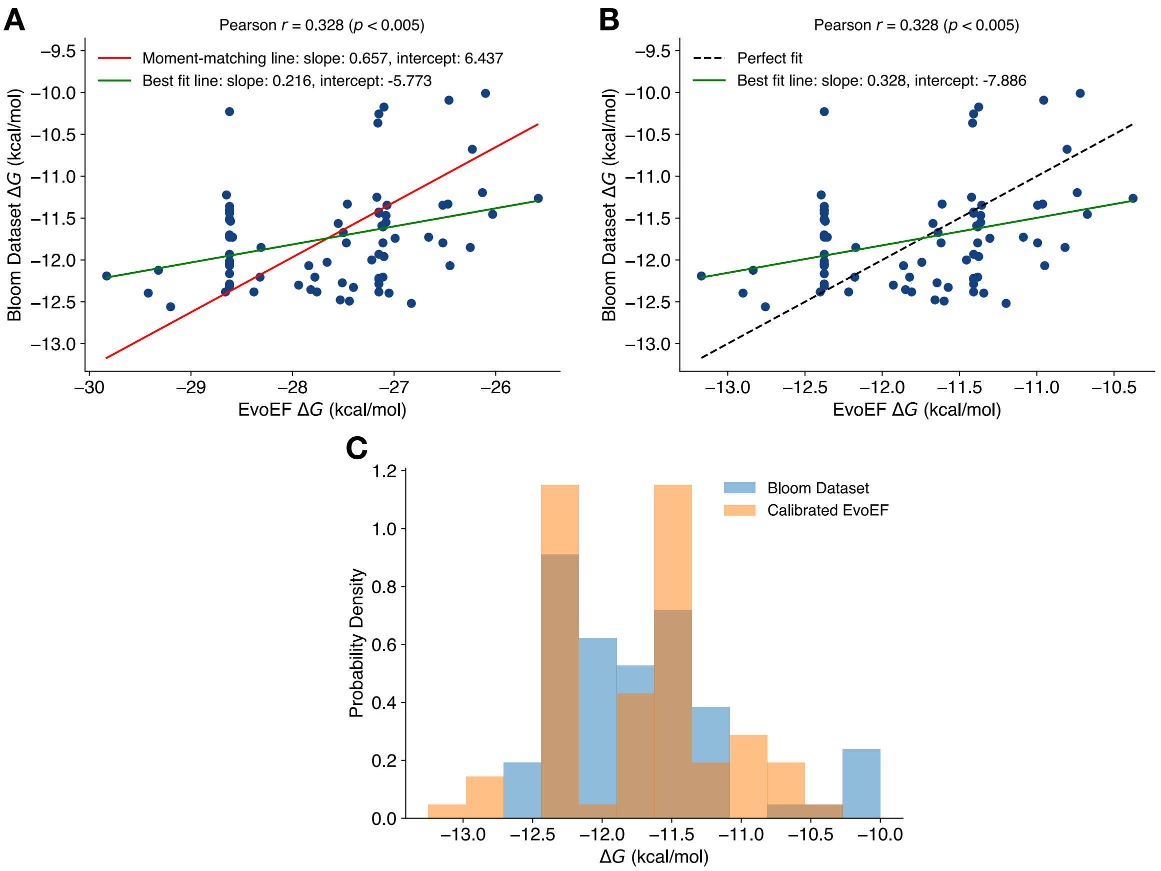

**Figure S3**: **Calibration of host-antigen force field binding affinities to experimental measurements**. (**A**) Force field and experimental binding affinities for the 77 antigens present in both datasets are positively correlated. (**B**) Calibration of force field binding affinities using moment-matching helps bring force field binding affinities absolutely closer to experimental ones. (**C**) The experimental data distribution and the calibrated force field data have matching means and variances.

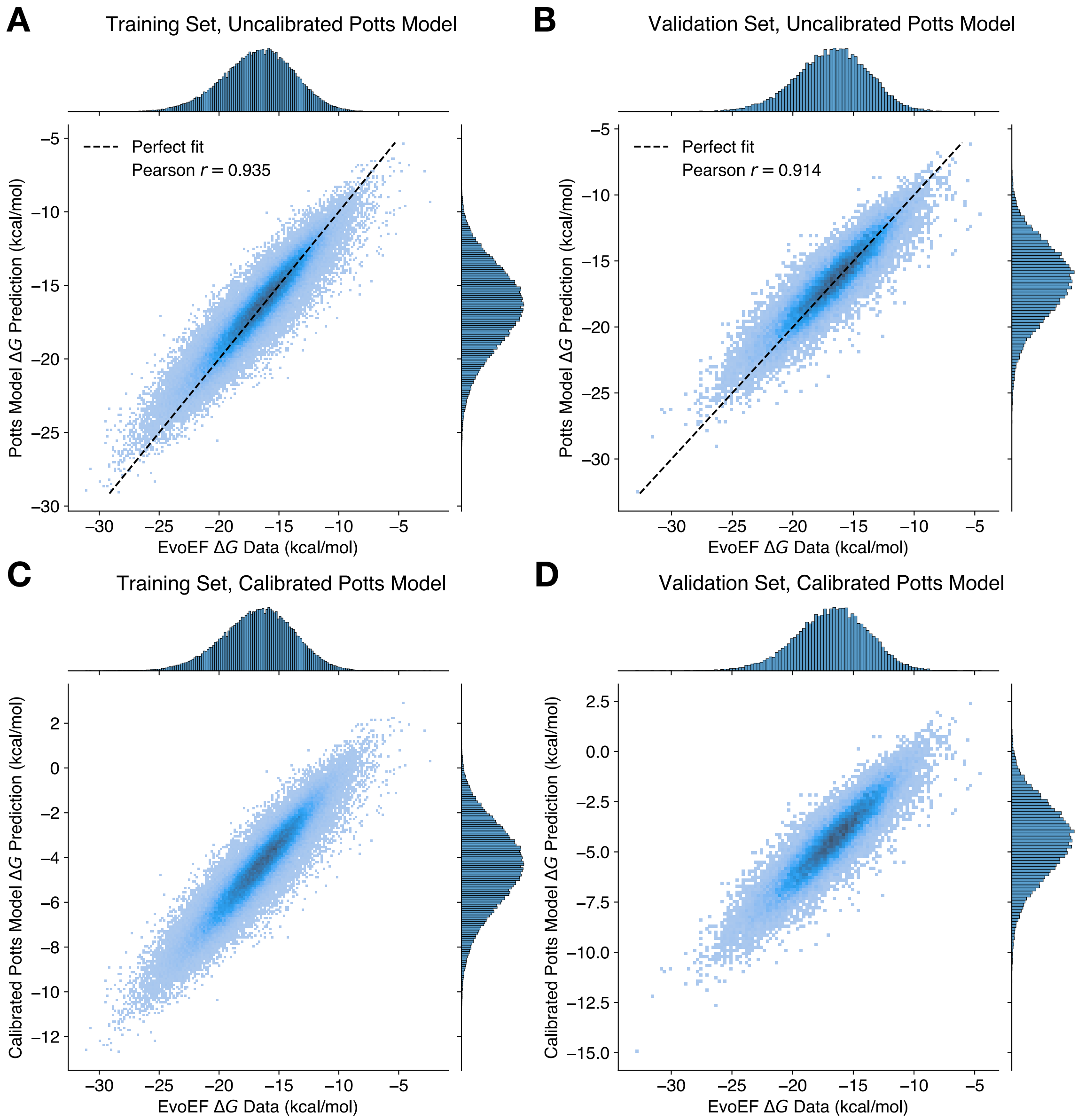

**Figure S4**: **Training, validation, and calibration (to experimental data) of Potts model for antibody-antigen binding affinities**. Potts model predictions on (**A**) training and (**B**) validation force field binding affinities show internal consistency. (**C**) and (**D**) show the same, but after calibration of the Potts model to experimental data.
