# Biophysical fitness landscape design traps viral evolution

**DOI:** 10.1101/2025.03.30.646233

**Published:** 2025-06-10

**Authors:** Vaibhav Mohanty, Eugene I. Shakhnovich

## Abstract

Rapidly evolving viruses mutate to escape antibodies generated by the human immune system, leading to periodic waves of infection and death. Modern vaccine design approaches typically focus on currently prevalent strains and thus are shortsighted and often rendered ineffective by emerging viral mutations. An ideal strategy for proactive vaccine design requires not only immediate effectiveness, but also control over future viral mutation trajectories and evolutionary fitness landscapes. Here, we introduce fitness landscape design with antibodies (FLD-A) to create optimal antibody repertoires to restrict viral fitness trajectories. By deriving a biophysical fitness model from microscopic chemical reactions, we show that while standard approaches to vaccination do not necessarily restrict evolution of viral escape, our systematic protocol can be used to design effective, proactive antibody repertoires to suppress the replication of future escape variants. Stochastic optimization is performed to discover an antibody ensemble which forces a viral surface protein to evolve according to user-defined fitness penalties against each strain, imposing a reshaped fitness landscape that disfavors viral proliferation. After validating the antibody-imposed fitness landscapes with *in silico* serial dilution experiments, we apply FLD-A to reshape the relative fitness effects of mutations within two SARS-CoV-2 genotype networks while maintaining overall fitness suppression. Finally, we introduce an iterative FLD-A protocol which consistently discovers vaccination target sequences that generate antibodies which simultaneously restrict wildtype and escape variant fitness trajectories. Biophysical FLD-A thus opens the door to evolutionary control and proactive vaccine, antibody, and peptide design, thinking several steps ahead of pathogen evolution.

## Full Text Availability

The license terms selected by the author(s) for this preprint version do not permit archiving in PMC. The full text is available from the preprint server.

