# Biophysical fitness landscape design traps viral evolution

**DOI:** 10.1101/2025.03.30.646233

**Published:** 2025-07-25

**Authors:** Vaibhav Mohanty, Eugene I. Shakhnovich

**Affiliations:** 1 Department of Chemistry and Chemical Biology, Harvard University, Cambridge, MA 02138; 2 Harvard/MIT MD-PhD Program, Harvard Medical School, Boston, MA 02115 and Massachusetts Institute of Technology, Cambridge, MA 02139; 3 Program for Health Sciences and Technology, Harvard Medical School, Boston, MA 02115 and Massachusetts Institute of Technology, Cambridge, MA 02139

## Abstract

Evolutionary adaptation is often visualized as a population’s stochastic climb toward the top of a fitness landscape. While there exist approaches to design or synthetically evolve proteins into desired structures, there is a lack of methodology for designing, tuning, and quantitatively reshaping the fitness landscapes themselves on which protein evolution takes place. Here, we introduce foundational principles of fitness landscape design (FLD) to customize the structural peaks and valleys of biophysical fitness landscapes with quantitative accuracy, offering robust control of long-term evolutionary outcomes. Our FLD algorithms use stochastic optimization of a chemically derived biophysical fitness model to consistently discover optimal antibody ensembles which force a target protein to evolve according to a user-specified target fitness landscape. We then apply FLD to suppress the fitnesses of two SARS-CoV-2 genotype neutral networks and to discover proactive vaccines that preemptively restrict escape variant fitness trajectories before they arise.

## 1 Introduction

**Figure 1:**
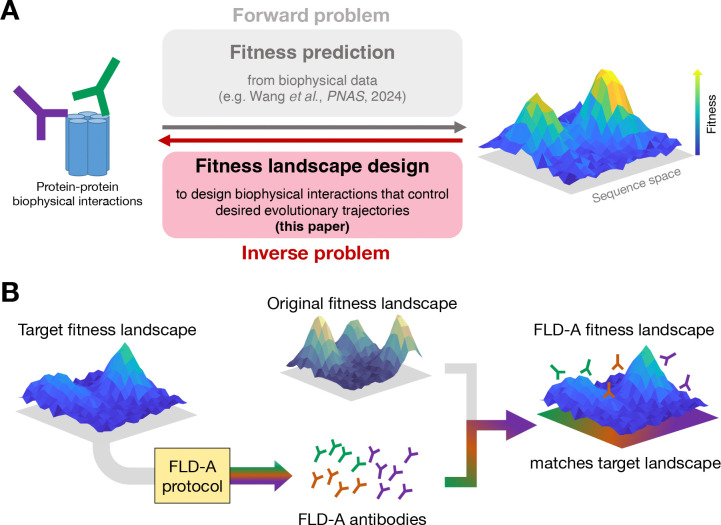
Overview of the fitness landscape design problem, and our computational solution, FLD-A, which using antibodies to quantitatively reshape fitness landscapes. (**A**) Many studies focus on the fitness prediction *forward* problem (gray), inferring fitness landscapes from biological data, including evolutionary time series data and biophysical interaction data, including our recent work on inferring SARS-CoV-2 fitness from host-antigen and antibody-antigen binding affinities^18^. In this paper, we introduce the *inverse* problem of *fitness landscape design* (red), where one uses a target fitness landscape to engineer biophysical interactions that influence protein evolution to take place according to the target landscape. (**B**) Our computational FLD protocols provide a solution to the fitness landscape design problem. Given a user-defined target fitness landscape for a particular target protein, the FLD-A algorithms presented in this work are used to discover an antibody ensemble which bind to the target protein and modify its experienced fitness landscape to match the target landscape. In the presence of the FLD-A antibodies, the target protein then would evolve according to the target landscape.

Fitness, in an evolutionary context, is a genotype’s (or phenotype’s) reproductive success, generally defined as a growth rate of a strain or species^1^. A *fitness landscape* ^2^ is the relationship between the set of all genotypes and their respective fitnesses, often visualized as a mountain-like structure^3–6^ (Figure 1A). In the 90+ years since their introduction by Sewall Wright^2^, fitness landscapes have proven to be a powerful tool for understanding evolutionary processes: for example, evolution can be viewed as a semi-random climb towards higher fitness peaks—a stochastic survival-of-the-fittest process^3,5,7^.

For proteins, it is well-established that biophysical interactions between amino acids can influence the shape of the fitness landscape and thereby affect evolutionary trajectories^8–14^. Many studies have accordingly focused on constructing fitness landscapes by fitting theoretical fitness models to biophysical data^6,15–18^ or directly through empirical observation of organism growth rates^3,19,20^. Construction of fitness landscapes from data represents an important “forward” problem in evolutionary biology because it uncovers possible evolutionary paths that are enabled by nature. However, most of these studies take the fitness landscape to be externally determined and fixed, focusing instead on how evolutionary dynamics would take place on that landscape. Quantitative control over the shape of the fitness landscape itself is not well-understood in the literature.

Here, we define and introduce the corresponding “inverse” problem, which we term *fitness landscape design* (FLD): for a user-chosen target fitness landscape, how can we control protein evolution to take place according to that target landscape (Figure 1A)? For instance, how is it possible to force protein sequences X and Y to cause an organism to replicate at rates $F_{X}$ and $F_{Y}$, respectively? In the present work, we explore the limits of fitness landscape designability and introduce computational protocols to solve the FLD problem by focusing on viral surface proteins and the design of fitness landscapes using antibodies that can suppress long-term viral fitness gains, offering solutions to open challenges that currently prevent proactive vaccine design.

Viruses constantly face pressure from human adaptive immunity to mutate, allowing the emergence of escape variants capable of increased replication and transmission, ultimately causing a greater number of human infections and deaths^21–24^. Even if vaccination efforts are rapid—like the development of the first SARS-CoV-2 mRNA vaccine^25^—by the time vaccines are adequately and widely deployed, viral escape mutations typically will have already emerged, seeding yet another wave of infections.

Current pandemic preparedness strategies involving vaccination are often shortsighted, focused on targeting today’s most prevalent antigens^26–28^ but can fail to account for tomorrow’s inevitable escape mutations^29^. Approaches which try to induce broadly neutralizing antibodies (bnAbs) to target multiple strains^30–33^ often face implementation challenges because they rely on targeting conserved but sterically inaccessible peptide regions^31–33^ or immunizing against too many strains at once, leading to B-cell “frustration” and poor immune response^31–33^. New strategies to sequentially expose the immune system to related antigens^33^ show promise in HIV-1^34^, but in influenza and dengue can cause “original antigenic sin” (OAS) to prevail—a phenomenon where the immune system remembers the original strain but ignores subsequent exposures to new strains, leading to outdated antibody generation and poor clinical outcomes^35–38^.

Modern vaccine design thus continues to lag one step behind viral evolution, and waves of infection due to endemic viruses such as influenza exhibit a vicious cycle, requiring frequent and periodic updates to develop a new vaccine each year^21–24^. Developing an approach to break this cycle requires not only improved foresight into future viral evolution, but also adequate *control* over the viral fitness landscape to reshape and ultimately trap its evolutionary trajectory in a low fitness state. Proactive vaccine design against viruses thus can map onto the FLD problem, specifically the problem of designing fitness landscapes that suppress long-term fitness growth of viral escape variants.

To solve the FLD problem, we develop a set of computational methods that discover antibodies that reshape viral protein fitness landscapes for optimal suppression of escape variants, which we call *fitness landscape design with antibodies* (FLD-A). After deriving a biophysical model of viral fitness from microscopic chemical reactions, we show that a systematic choice of antibody repertoire allows different viral surface protein mutants to be assigned new fitnesses with flexibility and independence. Upon application of these antibodies, the surface proteins then evolve according to the user-defined target landscape, as validated by *in silico* serial dilution experiments using microscopic chemical reaction dynamics simulations. We then introduce algorithms to design fitness landscapes for two application tasks: (1) molding the relative fitnesses of two SARS-CoV-2 neutral genotype networks while maintaining absolute fitness reduction and (2) discovery of vaccination targets which better trap the fitness trajectory of viral escape variants compared to typical vaccination, requiring only a single target antigen and without the need for sequential vaccination.

The FLD-A protocols, summarized in Figure 1B, open the door to proactive vaccine and antibody design^26–28,30,32,39^, offering promising new strategies for improving pandemic preparedness. Moreover, these computational methods can offer possible downstream applications to cancer therapeutics, such as reshaping cancer fitness landscapes with CAR-T cell^40,41^ design to disfavor immune escape, and small peptide drug design^42,43^. More broadly, the FLD-A fundamental open problem in evolutionary biology: controlling evolution to fight human disease.

## 2 Results

### 2.1 Biophysical model bridges fitness and binding affinities

We first develop a biophysical model that relates a viral surface protein sequence to the viral strain’s growth rate. We derive this model analytically by starting from microscopic chemical reactions between antigen, antibodies, and host proteins and mapping them onto absolute fitness. Consider 3 sets of chemical reactions: (1) the reversible binding of a viral protein sequence s to a host cell receptor, (2) the reversible binding of the antigen sequence s to an antibody paratope sequence $a_{n}$ which competitively inhibits antigen-host binding, and (3) the irreversible replication of the viral strain into many new copies via entry and eventual lysis of the host cell. In the Methods, we show how the kinetic rate equations for these reactions combine to directly equate to the *definition* of evolutionary fitness^1^, which then can be expressed mathematically:

(1) $$F(s)\approx k_{rep}\left(N_{o}-1\right)N_{ent}p_{b}(s),$$

where $k_{\text{rep}}$ is a single virion’s microscopic rate constant for cell entry and replication, $N_{o}$ is the average number of offspring produced by a single virion’s replication, $N_{\text{ent}}$ is the number of viral surface proteins used for host cell entry (i.e. fusion or spike proteins), and $p_{b}(s)$ is the probability that a single viral entry receptor with sequence s will be bound to the host receptors at equilibrium. This probability is given by

(2) $$p_{b}(s)\approx \frac{\left[H^{\text{total}}\right]e^{-\beta \Delta G_{H}(s)}}{C_{0}+\left[H^{\text{total}}\right]e^{-\beta \Delta G_{H}(s)}+\sum_{n}\;\left[{Ab}_{n}^{\text{total}\;}\left(a_{n}\right)\right]e^{-\beta \Delta G_{Ab}\left(s,a_{n}\right)}},$$

where $C_{0}$ is a reference concentration to set units, β is inverse temperature, $\Delta G_{H}(s)$ is host-antigen binding free energy, $\Delta G_{Ab}\left(s,a_{n}\right)$ is antigen-antibody binding free energy for the n-th antibody in the repertoire, $\left[H^{\text{total}}\right]$ is host receptor concentration, and $\left[{Ab}_{n}^{\text{total}}\left(a_{n}\right)\right]$ is the concentration of antibody with sequence $a_{n}$. Our biophysical fitness function now provides a direct genotype-fitness mapping from viral antigen sequence s to a fitness F(s).

**Figure 2:**
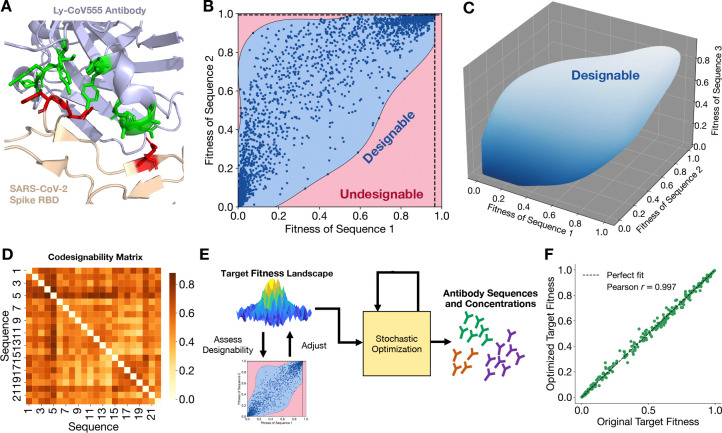
Fitness landscapes are designable and can be custom designed with stochastic optimization. (**A**) Antigen-antibody complex between SARS-CoV-2 RBD and Ly-CoV555, with residues subject to variation in this study for FLD highlighted in red and green, respectively. (**B**) Designability phase diagram for two antigen sequences, obtained by sampling random antibody ensembles and using an SVM to compute the designable region. Dark blue dots are the 10,000 randomly sampled fitness landscapes, each from a randomly generated antibody ensemble. The designable (light blue) region, obtained with an SVM, indicates possible fitness values that can be physically realized by some antibody ensemble, while the red region indicates inaccessible fitness assignments. Black dashed lines indicate the maximum possible fitness for each sequence, limited by host-antigen binding affinity, according to eq. (1). (**C**) 3D designability phase diagram for three antigen sequences, also obtained by sampling 10,000 random antibody ensembles and using an SVM to compute the designable region. (**D**) Codesignability matrix for a set of sequences, with higher codesignability scores indicating a larger designable region in the 2D phase diagram exemplified by (B). (**E**) Schematic for oFLD-A protocol, which outputs the antibody sequences and concentrations which realize the target fitness landscape. (**F**) Near-exact agreement between target fitness landscape and the fitness landscape realized by antibodies discovered by the oFLD-A protocol, with each green dot representing one of 256 different epitope sequences. *Note:* (**B**), (**C**), and (**F**) use normalized fitness, scaled down by $k_{rep}\left(N_{o}-1\right)N_{ent}$.

For concreteness, we focused on the biophysical fitness landscape of the SARS-CoV-2’s spike protein, for which we recently showed that a model similar to eq. (2) agrees with fitness measurements obtained from global PCR sequencing data^18^. We considered a set of mutable loci on the receptor binding domain (RBD) of the spike. We obtained Protein Data Bank (PDB) structures of the RBD bound to the human host receptor ACE2 which facilitates viral entry^44^ and the RBD bound to Ly-CoV555, a class 2 antibody^45^ which targets many of the same RBD amino acids involved in ACE2 binding^46,47^. To obtain binding free energies for mutated antigens and antibodies, we allowed amino acid variation at 4 SARS-CoV-2 antigen sites and at 11 Ly-CoV555 paratope sites (Figure 2A, Table S1). We performed EvoEF force field calculations^48^ to compute host-antigen binding free energies $\Delta G_{H}(s)$ which were then calibrated to experimental measurements^49^. For antibody-antigen binding free energy prediction, a Potts model was trained^50^ on $\Delta G_{Ab}(s,a)$ force field calculations and similarly calibrated (see Methods for details). These binding free energies were plugged directly into eq. (1) to obtain fitness F(s) for SARS-CoV-2 variant sequence s in the presence of any chosen antibody ensemble.

### 2.2 Fitness landscapes are designable

Now, with an easily computable genotype-fitness mapping, we assessed the designability of the fitness landscape: for instance, if genotype 1 has low fitness, how low or high can genotype 2’s fitness be? For the simplest case of two randomly chosen SARS-CoV-2 antigen sequences, the space of all possible fitness assignments is shown in Figure 2B. By generating random ensembles of antibodies (see Methods), we discovered what subset of fitness assignments are practically realizable by some antibody repertoire (the blue, “designable” region in Figure 2B outlined using a support vector machine, or SVM), and what fitness assignments are not realizable by any antibody repertoire (the red, “undesignable” region). Together, the two regions produce a “phase diagram” for fitness landscape designability. This diagram can certainly be generalized to more than two sequences (Figure 2C), though visualizations become difficult beyond three dimensions.

For a pair of sequences, when the designable region’s area—which we call the *codesignability score*—is larger, it means that two genotypes’ fitnesses are more independent of each other, so there is a broader range of fitness choices for those sequences. The codesignability matrix (Figure 2D) captures codesignability scores for all pairs of sequences in a set, which lets us see which sequences have more flexibility in fitness assignment. We will show in a subsequent application that the codesignability matrix can capture useful biophysical information about neutral genotype networks.

Since codesignability scores are generally greater than zero, we can manually reshape fitness landscapes for a set of antigenic sequences by finding an appropriate antibody ensemble. Concretely, our goal is to take some user-defined target fitness landscape $F_{\text{target}}(s)$, chosen from the designable region, and find an antibody ensemble that causes the viruses to actually evolve according to that target landscape. To match the biophysical fitnesses F(s) from eq. (1) to the target fitnesses $F_{\text{target}}(s)$, we perform simulated annealing^51^ with the Metropolis-Hastings algorithm^52^ to update antibody sequences and concentrations, progressively minimizing the least-squares loss between the target landscape $F_{\text{target}}(s)$ and biophysical landscape F(s) by iteratively updating the antibody concentrations and paratope amino acids. The output of this optimization FLD-A (oFLD-A) protocol, summarized in Figure 2E, is a list of antibody variants and their respective concentrations which would force the viruses to experience the target fitness landscape. Although we visualize the designability phase diagrams for only two or three sequences, as an illustrate example we test the oFLD-A protocol on a set of 256 antigen sequences and find that the protocol successfully and reproducibly obtains an antibody ensemble for which biophysical fitnesses nearly perfectly match the target fitnesses (Figure 2F). Thus, we have shown that custom design of fitness landscapes is indeed possible.

### 2.3 *In silico* serial dilution experiments with chemical reaction dynamics validate designed fitness landscapes

In the presence of the designed antibody repertoire, we next wanted to see if viral variants would actually evolve according to the target fitness landscape in dynamical settings. To validate FLD-A, we performed a series of *in silico* serial dilution experiments, modeled after standard *in vitro* procedures^6^, to allow strain-versus-strain competition between viral variants.

**Figure 3:**
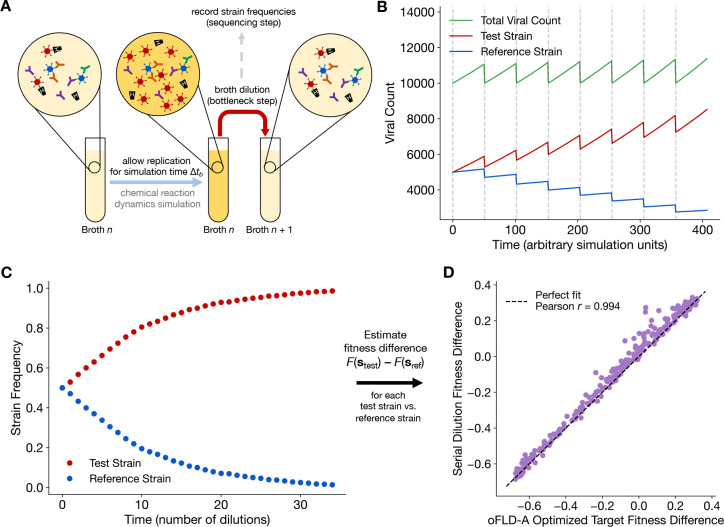
*In silico* serial dilution experiments validate FLD-A. (**A**) Schematic of *in silico* serial dilution experiments using microscopic chemical reaction dynamics. (**B**) Time evolution of viral counts from chemical reaction dynamics across multiple broth dilution steps. (**C**) Observable strain frequency data, measured at the broth dilution steps indicated by gray dashed lines in (A) and (B), representing the data available to an experimenter with no knowledge of the underlying microscopic chemical reactions. (**D**) Near-exact agreement between optimized target fitness difference (equivalent to vertical axis from Figure 2F) and simulation fitness difference. Plotted fitnesses are normalized, scaled down by $k_{\text{rep}}\left(N_{o}-1\right)N_{\text{ent}}$.

Using the BioNetGen biochemical reaction network modeling software^53^, we simulated the time-dependent chemical reaction dynamics given by the microscopic binding and replication reactions previously used to build the biophysical fitness model (see Methods for exact reactions). For the 256 sequences for which we designed the fitness landscape, we conducted 255 *in silico* experiments. Labeling one sequence as the reference strain, each of the remaining 255 strains separately faced the reference strain in a head-to-head competition over the course of many broth dilutions (Figure 3A). In each experiment, within a broth the two viral strains experienced exponential growth at different rates causing the total viral count to form a prototypical sawtooth-like wave^54^ over the course of many broth dilutions (Figure 3B). At each broth dilution/transfer step, the relative frequencies of the two viral strains were recorded, akin to sequencing *in vitro* samples.

In a laboratory setting, an experimenter would validate the designed fitness landscape by measuring fitness directly from the population-genetic strain frequency data, without chemical measurements of binding affinities, and compare independently obtained population-genetic “observed” fitnesses to the designed biophysical fitnesses. To obtain such observations for our *in silico* experiment, we use the time series of recorded strain frequencies (Figure 3C) to extract the fitness difference $F\left(s_{\text{test}}\right)-F\left(s_{\text{ref}}\right)$ between a test strain $s_{\text{test}}$ and reference strain $s_{\text{ref}}$ (see Methods for details). Repeating the procedure for the remaining 254 test strains provided a complete observed fitness landscape, where the observed landscape was obtained purely from genetic frequency data without knowledge of the microscopic chemistry.

In Figure 3D, we plot the observed fitness landscape versus the optimized target fitness landscape expected from the designed antibody repertoire (the vertical axis from Figure 2F). Not only do we obtain impressive Pearson correlation r=0.994, but fitness is predicted with near-exact quantitative accuracy including scaling prefactors, a merit of the biophysical fitness formula eq. (1). Combined with our published *in vitro* ^6^ and genomic^18^ evidence to support the validity of fitness functions similar to eq. (2) (which until now had been conjectured but not mathematically justified), our *in silico* serial dilution experiments here provide compelling evidence that viruses do evolve according to fitness landscapes designed with eq. (1). We now use eq. (1) directly for applied design tasks.

### 2.4 Tuning the fitness landscape of two SARS-CoV-2 neutral genotype networks with customizable fitness penalties

**Figure 4:**
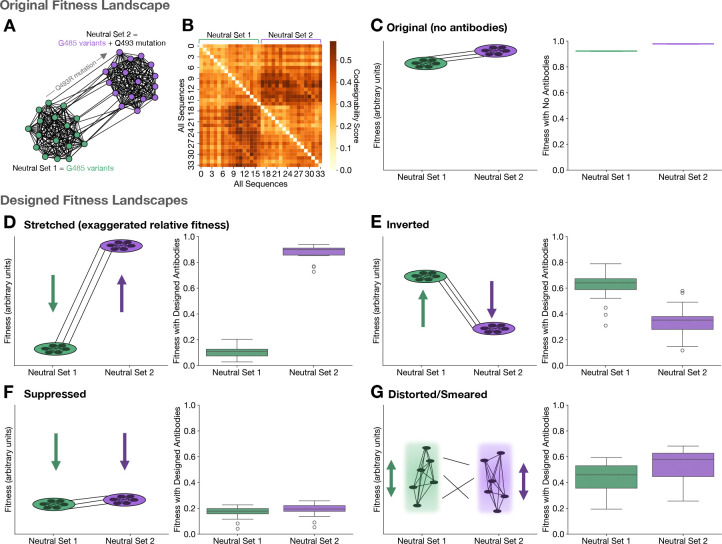
SARS-CoV-2 neutral networks are tunable. (**A**) Two SARS-CoV-2 neutral networks, each with 17 genotypes variable at residue G485, connected by the fitness-increasing mutation Q493R. (**B**) Codesignability matrix of the 34 genotypes in the 2 neutral networks, showing general trend of codesignability between neutral sets 1 and 2. (**C**) Fitnesses of original neutral networks. (**D**) Stretching relative fitnesses of the neutral sets using oFLD-A, making neutral set 2 even more fit relative to neutral set 1. (**E**) Inverting the relative fitnesses of the neutral sets using oFLD-A, turning Q493R from a fitness-increasing mutation into a deleterious one. (**F**) Suppressing the fitnesses of both neutral sets using oFLD-A, approximately maintaining their relative fitnesses but decreasing absolute fitness. (**G**) Distorting the two neutral networks using oFLD-A, breaking the neutrality of the networks altogether. Left-hand panels in (**C-F**) show schematics while right-hand panels show box plots of the 34 fitnesses after applying the oFLD-A protocol.

We now apply the oFLD-A protocol to design the fitness landscape of two SARS-CoV-2 neutral genotype networks while maintaining antiviral activity. The SARS-CoV-2 wildtype strain has 16 possible mutations at residue G485 that result in zero change in fitness. These 17 genotypes (including the wildtype) are all single mutational neighbors of each other, and are referred to as a neutral set, neutral network, or genotype network^55–58^. The Q493R mutation increases fitness of these 17 genotypes without breaking neutrality; it naturally occurred between the wildtype and Omicron variants of SARS-CoV-2^59^. Thus, the 17 Q493R− and 17 Q493R+ variants at the G485 site form two neutral networks which are interconnected fitness plateaus (Figure 4A).

We computed the codesignability matrix for these 34 genotypes and found that the matrix separates into a 2 × 2 block matrix, with the off-diagonal blocks generally darker than the on-diagonal blocks (Figure 4B). This indicates that the two neutral networks, overall, should be codesignable as groups, and sequences within a neutral network are expected to be less codesignable. In Figure 4C, we plot a schematic representation of the two neutral networks as well as their actual fitness distributions, which are flat by definition.

Now, we demonstrate how oFLD-A can transform the original fitness landscape into a wide range of user-specified designs, while maintaining overall absolute fitness reduction. Rescaling fitnesses to a range between 0 and 1 for convenience, we set a fitness target of 0 for the Q493R− neutral set (neutral set 1) and a target of 1 for the Q493R+ neutral set (neutral set 2). The oFLD-A protocol then generates an antibody ensemble which dramatically exaggerates the relative fitness differences between the 2 neutral networks, dropping the fitness of neutral set 1 much more than the fitness of neutral set 2 (Figure 4D). By setting a fitness target of 1 for neutral set 1 and a target of 0 for neutral set 2, we can flip the relative fitnesses of the two neutral sets, causing neutral set 2 genotypes to generally have lower fitness than neutral set 1 genotypes (Figure 4E). Q493R can thus be transformed from a fitness-increasing mutation into a deleterious mutation.

By setting fitness targets to 20% of their original values, we suppress the fitnesses of both neutral sets (Figure 4F). Lastly, we can break the neutrality of both neutral sets by assigning completely random fitnesses chosen from a uniform interval to all 34 genotypes, distorting the fitness landscape entirely (Figure 4G).

FLD-A thus provides a method for molding fitness landscapes like clay: stretching, inverting, suppressing, and distorting the landscape are made possible through the optimization protocol introduced previously. We emphasize that, according to eq. (1), all of these transformations only lower—never increase—fitnesses since the maximum fitness of a strain is determined by its host receptor binding ability, which is not controlled by antibodies. This simultaneous retunability of relative fitness landscapes while suppressing absolute viral fitness makes effective and safe vaccine design possible with FLD-A.

### 2.5 Iterative FLD-A discovers proactive vaccines that trap viral fitness trajectories

**Figure 5:**
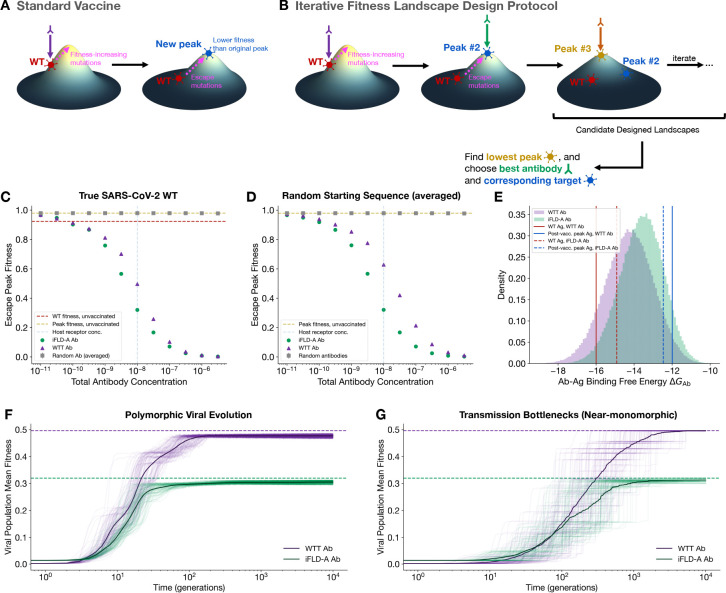
Restricting the peak fitness of viral escape variants. (**A**) Standard vaccination protocol, generating the wildtype-targeting antibody, targets the wildtype sequence. Viral escape mutations cause fitness increases towards the post-vaccination fitness peak. (**B**) iFLD-A finds the vaccine target which minimizes post-vaccination peak fitness through an iterative procedure of targeting the new global fitness peak in the context of the previous antibody by searching the antigenic sequence space and computing fitness using the biophysical model in eq. (1). (**C**) Comparison of iFLD-A, standard vaccination (WTT antibody), and random antibodies, using the SARS-CoV-2 wildtype as the starting sequence. (**D**) Same as (**C**) but averaged over random starting sequences. (**E**) iFLD-A and standard vaccination antibodies’ binding affinities to both target antigens. (**F**) Polymorphic and (**G**) near-monomorphic viral evolutionary dynamics demonstrate that populations exhibit slower fitness growth and are trapped under a lower fitness ceiling by the iFLD-A antibody compared to the WTT antibody. Dashed lines indicate global maximum fitness, and translucent lines are population mean fitness trajectories from each of 100 Wright-Fisher trials.

We now apply FLD-A to vaccine design to create evolutionary traps that restrict viral fitness trajectories. According to eq. (1), in the absence of any antibodies, viral fitness can increase by improving host receptor binding, mutating along the fitness landscape toward the fitness peak. After vaccinating against the wildtype or most prevalent strain, this fitness peak can shift (Figure 5A). This immune pressure encourages escape mutations, which decrease antibody-antigen binding affinity.

Given the propensity for escape mutations to occur, we developed a proactive vaccination protocol that plans *ahead*, analogous to a chess player calculating future moves. The goal of our iterative FLD-A (iFLD-A) protocol is to find a vaccine target antigen which optimally suppresses the *post-vaccination* fitness peak (Figure 5B), so that escape mutants’ fitnesses are reduced as much as possible.

We first vaccinate against the wildtype/starting strain, obtaining a high-affinity antibody (the wildtype-targeting, or WTT, antibody) to this original strain. We call this the “standard vaccination” protocol. Next, we exhaustively search over the the space of 20^4^ = 160,000 antigenic sequences to discover the new fitness peak (“Peak #2”) in the presence of the WTT antibody, which is the escape variant with the highest fitness in the presence of the first antibody. Vaccinating against this peak escape variant generates “Antibody #2.” The peak-fitness escape variant in the presence of Antibody #2 is then targeted next. We continue to iterate this procedure until the new global fitness peak maps back onto a previously discovered one or until a maximum number of iterations is reached. Lastly, the protocol identifies the lowest fitness peak, which immediately reveals the ideal antibody and target antigen for fitness peak suppression (Figure 5B). Vaccinating against this target antigen would then place a low ceiling on escape variants’ fitness, generally reducing escape variant fitness more than standard vaccination would. Unlike vaccines updated *reactively* in response to newly emergent variants, restricting the fitness trajectory by vaccinating against the iFLD-A target antigen offers a *proactive* means to suppress viral proliferation.

To evaluate performance, we calculated the impact of the iFLD-A antibody, the WTT antibody, and random antibodies on the post-vaccination global fitness peak, at fixed antibody concentrations (Figure 5C). In general, the iFLD-A antibody consistently outperformed the WTT antibody and random antibodies in reducing the peak fitness of escape variants (Figure 5C; extended results in Figure S1A). To see if the same phenomenon would hold if the SARS-CoV-2 wildtype were different (i.e. if the pandemic had begun with a different starting sequence), we repeated the same study for starting sequences chosen at random. Averaging over an approximately uniform distribution of starting fitnesses (see Methods), we found similar results, with the iFLD-A antibody reducing the escape fitness peak more than the WTT antibody and random antibodies (Figure 5D). As expected, these differences in fitness reduction became less prominent in the limits of very high or very low antibody concentration, aside from small fluctations.

To understand why the iFLD-A vaccination targets generally work better than the standard vaccination target, we compared the binding free energies of the two antibodies to each protocol’s target antigens. Considering the SARS-CoV-2 wildtype case, we found that the iFLD-A antibody binds more strongly to the iFLD-A target antigen than the WTT antibody does (dashed and solid blue lines in Figure 5E; Figure S1B). The tradeoff is that the iFLD-A antibody has to sacrifice some binding affinity to the wildtype strain (dashed and solid red lines in Figure 5E; Figure S1C). Fortunately, this sacrifice is often small enough to ensure that the iFLD-A antibody still substantially suppresses wildtype fitness, often comparable to standard vaccination (Figure S1D). The iFLD-A protocol thus achieves a lower post-vaccination peak fitness with limited sacrifice of wildtype neutralization—the best of both worlds.

To ensure that the new vaccination protocol would not inadvertently lead to escape variants with tighter host-binding affinity, we calculated host binding free energies of the post-vaccination global peaks from both protocols. We observed that vaccination by either iFLD-A or WTT always forced escape variants to become weaker binders to host receptors compared to the global fitness peak with no antibodies (Figure S1E). Furthermore, since the iFLD-A antibody does not directly target the wildtype strain, we also wanted to confirm that the wildtype strain does not become an escape variant under the new vaccination protocol. Empirically, we found that for antibody concentrations ≥ 10^−9^, the iFLD-A antibody decreased peak escape fitness more than the WTT antibody ≥ 97.5% of the time, and for antibody concentrations ≤ 10^−9.5^, the iFLD-A antibody decreased peak escape fitness more than the WTT antibody between 75.5% and 93.5% of the time. Thus, with only the iFLD-A antibody, the escape peak fitness is maximally suppressed almost all of the time, and only in the worst case the iFLD-A returns the same antibody as the standard vaccination.

Finally, we simulated viral escape dynamics using the Wright-Fisher model (Methods) in the presence of the WTT antibody or the iFLD-A antibody both in the polymorphic regime ($N_{\text{pop}}\mu L_{\text{Ag}}\gg 1$), corresponding to large viral populations $N_{\text{pop}}$ with high mutation rate per site μ^6^, and near the monomorphic regime $\left(N_{\text{pop}}\mu L_{Ag}\ll 1\right)$^5^, representing small infective populations in transmission events. We found that in both polymorphic (Figure 5F and Figure S2A) and near-monomorphic (Figure 5G and Figure S2B) regimes, populations exhibited slower mean fitness growth and were trapped under a lower fitness ceiling by the iFLD-A antibody than the WTT antibody.

Together, these results suggest that at the start of the COVID-19 pandemic, vaccinating against the target strain identified by iFLD-A would have led to a lower ceiling on the fitness of escape variants, effectively trapping the fitness trajectory of escape variants below a lower fitness threshold. By vaccinating against a future global peak by anticipating escape mutations, the iFLD-A protocol generates a tighter-binding antibody to future escape variants with little sacrifice to the neutralization capacity of the wildtype or current dominant variant.

## 3 Discussion

The FLD-A algorithms represents an advance toward addressing a fundamental inverse problem in evolutionary biology: the control of evolutionary outcomes via fitness landscape design, which has wide repercussions for improving pandemic preparedness and the treatment of any rapidly evolving human disease-causing agent, including microbial pathogens and cancers. In this work, we showed that a biophysical fitness function for viral surface proteins could be used in a stochastic optimization protocol to design antibody repertoires which achieve user-defined target fitness landscapes, thus allowing quantitative control over protein evolution.

FLD-A offers a new perspective on vaccine design. Modern rational design of epitope-targeting vaccines relies on finding high affinity antibodies for specific prevalent antigens^26–28^ or on generating broadly neutralizing antibodies through antigen ensembles or sequential vaccination^30,32^, which risks B cell frustration or original antigenic sin. However, we show with the iFLD-A protocol that even a single antibody directed against a prudent choice of target antigen can impose post-vaccination fitness thresholds on escape variants along with wildtype neutralization. Our proactive vaccine design approach thus offers a potential solution for both B cell frustration and original antigenic sin because iFLD-A does not require multiple target antigens to induce cross-reactive antibodies, nor does it require sequential vaccination. Instead, a single carefully chosen target antigen can potentiate both wildtype and escape variant neutralization. Though we focus on a single epitope in this study, FLD-A can easily be scaled to multiple epitopes by modifying eq. (1). Our forthcoming work will extend the serial dilution-based fitness model presented here to *in vivo* settings where immune cells are actively involved in viral clearance. Additionally, to address larger epitope sizes, the iFLD-A protocol can be extended by replacing exhaustive fitness peak searches with gradient-based stochastic optimization algorithms enabled by continuous space protein sequence representations^60^.

Additionally, this work sets the stage for many directions of applications and advancements stemming from the FLD-A protocols presented here. In addition to *in vitro* serial dilution experiments, future directions for further developing FLD-A include epidemiological modeling of iFLD-A vaccine effects, using deep learning and large language models for binding affinity prediction^18,61,62^, small peptide drug design^42,43^, designing evolutionary fitness traps for cancers through CAR-T cell receptor design^40,41^, and more. Fitness landscapes are no longer simply visual aids or theoretical tools^3–6^ as they have been for nearly a century^2^, but rather are entities designable using biophysical principles that are capable of controlling longer-term evolutionary outcomes to fight pathogens.

## 4 Methods

### 4.1 Biophysical fitness from microscopic chemical reactions

In this model, we consider a well-mixed solution of virions, antibodies, and host cells. As in the main text, we denote the viral strain as s, the chemical species referring to the entire virion of that strain as V(s), and the chemical species referring to that strain’s surface protein (fusion or spike) responsible for host cell entry as $V_{\text{ent}}(s)$. Similarly, the relevant subset of the antibody sequence (the paratope) is called a, with the n-th type of antibody in the ensemble with paratope $a_{n}$ called ${Ab}_{n}\left(a_{n}\right)$. Host cell receptors are called H, concentrations of any chemical species X are denoted by the usual brackets [X], and two bound chemical species X and Y are denoted as X·Y.

The definition of absolute fitness F(s) for a viral strain s is the baseline replication rate of the virus

(M1) $$\frac{d[V(s)]}{dt}\equiv F(s)[V(s)].$$

Our model starts with three chemical reactions: the host-antigen binding reaction,

(M2) $$V_{ent}(s)+H\underset{K_{d,H}(s)}{⇌}V_{ent}(s)\cdot H,$$

with dissociation constant $K_{d,H}(s)$, the antibody-antigen binding reaction

(M3) $$V_{ent}\left(s\right)+{Ab}_{n}\left(a_{n}\right)\underset{K_{d,Ab}\left(s,a_{n}\right)}{⇌}V_{ent}\left(s\right)\cdot {Ab}_{n}\left(a_{n}\right),$$

with dissociation constant $K_{d,Ab}\left(s,a_{n}\right)$, and the replication reaction for a virion bound to j host receptors

(M4) $$V(s)\cdot (jH)\overset{j\;k_{rep}\;}{\to }N_{o}\;V(s),.$$

where $k_{\text{rep}}$ is the baseline rate of cell entry and replication via each of the j receptors, and $N_{o}$ is the number of offspring produced. From these reactions, we calculate (see Supplementary Note 1 for full derivation) that

(M5) $$\frac{d[V(s)]}{dt}\approx k_{rep}\left(N_{o}-1\right)N_{ent}p_{b}(s)[V(s)],$$

where $N_{\text{ent}}$ is the number of viral entry proteins on the surface, and $p_{b}(s)$ is the approximate fraction of viral entry proteins which are bound to host receptors at equilibrium:

(M6) $$p_{b}(s)\approx \frac{\left[H^{\text{total}}\right]e^{-\beta \Delta G_{H}(s)}}{C_{0}+\left[H^{\text{total}}\right]e^{-\beta \Delta G_{H}(s)}+\sum_{n}\;[{Ab}_{n}^{\text{total}}\left(a_{n}\right)]e^{-\beta \Delta G_{Ab}\left(s,a_{n}\right)}},$$

with $C_{0}$ being a reference concentration to set units, β being inverse temperature, and $\Delta G_{H}(s)=\beta^{-1}log\;K_{d,H}(s)$ as well as $\Delta G_{Ab}\left(s,a_{n}\right)=\beta^{-1}log\;K_{d,Ab}\left(s,a_{n}\right)$ being binding affinities. The result from eq. (M5) is matched with the definition of absolute fitness in eq. (M1) to provide a *free energy-fitness relation*:

(M7) $$F(s)\approx k_{rep}\left(N_{o}-1\right)N_{ent}p_{b}(s),$$

which was presented in the Results section.

### 4.2 Fitness calculations

#### Force field binding free energy calculations.

We obtained PDB structures for the antigen-antibody complex (PDB number 7KMG) and antigen-host complex (PDB number 7DQA). 4 antigen residues, 5 antibody light chain residues, and 6 antibody heavy chain residues (Table S1) were chosen to be the mutable residues for fitness landscape design. Each selected antigen residue was involved in contact with both the antibody and the host, defined by < 8 Å distance between $C_{\alpha }$ atoms. We exhaustively computed host-antigen binding free energies $\Delta G_{H}(s)$ for all 20^4^ = 160, 000 antigenic variants (i.e. all possible amino acid sequences across the 4 antigenic sites) using EvoEF force field^48^ calculations performed at default (standard) temperature, consistent with calculation of β in eq. (M7) and BioNetGen simulations.

#### Potts model for antibody-antigen binding free energy.

For the antigen-antibody complex, mutations could occur at the 4 antigenic sites and at all 11 antibody sites, so with 20^4+11^ ≈ 3.28 × 10^19^ possible variants, exhaustive free energy calculation is impossible. So, we randomly sampled 160,000 variants and computed $\Delta G_{Ab}(s,a)$ using EvoEF. We used these data to train the parameters of a site-symmetric, spin-asymmetric chiral Potts model:

(M8) $$\Delta G_{Ab}^{Potts}(\sigma )=G_{0}+\sum_{i=1}^{4}h_{i}\left(\sigma_{i}\right)+\sum_{i=1}^{4}\sum_{j=1}^{4}\left[J_{ij}\left(\sigma_{i},\sigma_{j}\right)+J_{ji}\left(\sigma_{i},\sigma_{j}\right)\right],$$

where σ is a length 15 vector combining antigen sequence s and antibody sequence a. Using a 80%−20% training-validation split, we used stochastic gradient descent to train the 90,301 parameters $G_{0}$, $\left\{h_{i}\left(\sigma_{i}\right)\right\}$, and $\left\{J_{ij}\left(\sigma_{i},\sigma_{j}\right)\right\}$. The Potts model achieved internal consistency, with training Pearson r=0.935 (Figure S4A) and validation r=0.914 (Figure S4B).

#### Calibrating free energy predictions to experimental data.

Starr et al.^49^ conducted a deep mutational scan across the SARS-CoV-2 spike RBD and reported binding free energies. Since their mutations were not fully combinatorial, we extracted the 77 sequences in their dataset which overlapped with our antigenic sequences (19 single-site mutations at each of the 4 antigenic sites, plus the wildtype). Experimental $\Delta G_{H}(s)$ measurements are plotted against the force field calculations in Figure S3A. We calibrated the force field calculations to experimental measurements by moment-matching. By computing the means $\mu_{\Delta G}$ and standard deviations $\sigma_{\Delta G}$ of both force field and experimental overlapping datasets, we applied the linear transformation

(M9) $$\Delta \tilde{G}=\left[\mu_{\Delta G_{\text{experiment}}}-\mu_{\Delta G_{\text{force}\;\text{field}}}\left(\frac{\sigma_{\Delta G_{\text{experiment}}}}{\sigma_{\Delta G_{\text{force}\;\text{field}}}}\right)\right]+\frac{\sigma_{\Delta G_{\text{experiment}}}}{\sigma_{\Delta G_{\text{force}\;\text{field}}}}\Delta G_{\text{force}\;\text{field}}$$

to obtain the calibrated $\Delta {\tilde{G}}_{H}(s)$ force field binding free energies (Figure S3B). This calibration allows the means and standard deviations of the overlapping data distribution to match between force field and experiment (Figure S3C). The same calibration equation was then applied to the antibody-antigen Potts model (Figure S4C and D).

#### Fitness from free energy calculations.

Throughout this work, calibrated $\Delta {\tilde{G}}_{H}(s)$ and calibrated Potts model-predicted $\Delta {\tilde{G}}_{Ab}^{\text{Potts}}(s,a)$ were plugged into eq. (M7) to compute fitness.

### 4.3 Computing fitness landscape designability phase diagram and codesignability matrix

To compute a fitness landscape designability phase diagram and codesignability matrix, we randomly sampled the space of antibody ensembles. Antibodies were initialized with random paratope sequences at the 11 mutable sites, and starting concentrations were initialized over a broad range of order of magnitudes, heuristically adjusting the range to ensure that all fitnesses were not all close to 0 or all close to maximal. We sampled 10,000 sets of random antibody ensembles to produce the results in Figure 2B and C, where we used 3 antibodies, and for Figure 4B, where we used 4 antibodies. For any sequence pair (or triplet), we used a support vector machine (SVM) with a radial basis function kernel to identify a decision boundary separating designable and undesignable regions. This was implemented with Scikitlearn’s^63^ OneClassSVM, with parameters γ=5 (γ=6 for the 3D designability diagrams) and ν=0.0001. Codesignability scores for sequence pairs were calculated by splitting the unit square into a 100 × 100 grid on which the SVM decision was evaluated, and then summing the area of the squares where the decision was positive.

### 4.4 Stochastic optimization for fitness landscape design with antibodies (oFLD-A protocol)

The oFLD-A protocol uses a mean-square-error (MSE) loss (multiplied by a factor of 10,000 for numerical convenience) between the biophysical fitness landscape $F_{\text{target}}(s)$ from eq. (M7) and a target fitness landscape $F_{\text{target}}(s)$:

(M10) $$ℒ\left[F(s)|F_{\text{target}}(s)\right]\propto \sum_{s}{\left(F(s)-F_{\text{target}}(s)\right)}^{2}.$$

We initialized random antibody ensembles by picking random amino acids at the mutable positions. We then picked random starting concentrations which were some random number between 0 and 1 multiplied by $10^{\eta^{*}}$, where $\eta^{*}$ is the exponent which minimized the loss for that starting set of random residues; for Figure 4E, we instead initialized with a remapped $\eta^{*}\mapsto \eta^{*}+1$ because this tended to improve convergence to a deeper minimum.

We then perform simulated annealing, using the Metropolis-Hastings algorithm^52^, with an exponential cooling schedule starting with simulation temperature $T_{\text{MCMC}}=10$ and dividing by a factor of 10 after every 10,000 time steps until the 10,000 steps at the final simulation temperature (0.1 or 0.01) were completed. At each time step, we first proposed the swap of a random mutable residue of a random antibody to a random amino acid. The scaled MSE loss was recalculated, and the proposal was accepted or rejected with the standard Metropolis acceptance probability

(M11) $$A(\left\{a_{n}\right\},\left\{\left[{Ab}_{n}\left(a_{n}\right)\right]\right\}\to \{a_{n}^{\text{update}}\},\left\{\left[{Ab}_{n}\left(a_{n}\right)\right]\right\})\;=min\left(1,e^{-\frac{10,000}{T_{\text{sim}}}({ℒ}_{\text{MSE}}(\{a_{n}^{\text{update}}\},\left\{\left[{Ab}_{n}\left(a_{n}\right)\right]\right\})-{ℒ}_{\text{MSE}}\left(\left\{a_{n}\right\},\left\{\left[{Ab}_{n}\left(a_{n}\right)\right]\right\}\right))}\right)$$

We then proposed a concentration update by randomly increasing or decreasing each concentration by $10^{\eta^{*}-1}C_{0}$ if the simulation temperature was $T_{MCMC}\in \{10,\;1,\;0.1\}$ or by $10^{\eta^{*}-2}C_{0}$ if $T_{\text{MCMC}}=0.01$. The concentration update was accepted with probabiltiy

(M12) $$A(\left\{a_{n}\right\},\left\{\left[{Ab}_{n}\left(a_{n}\right)\right]\right\}\to \left\{a_{n}\right\},\{\left[{Ab}_{n}^{\text{update}}\left(a_{n}\right)\right]\})\;\;=min\left(1,e^{-\frac{10,000}{T_{\text{sim}\;}}\left({ℒ}_{\text{MSE}}(\left\{a_{n}\right\},\{\left[{Ab}_{n}^{\text{update}}\left(a_{n}\right)\right]\})-{ℒ}_{\text{MSE}}\left(\left\{a_{n}\right\},\left\{\left[{Ab}_{n}\left(a_{n}\right)\right]\right\}\right)\right)}\right).$$

The oFLD-A algorithm thus uses alternating antibody sequence and concentration updates to find an ideal ensemble for matching the target fitness landscape.

### 4.5 *In silico* serial dilution experiments using chemical reaction dynamics

We used the “bionetgen” Python package to generate BioNetGen^53^ simulation files for each serial dilution experiment. For BioNetGen’s ODE simulator, concentrations use arbitrary units, as do replication rate constants; the time scale of simulations is set by the rate constants. The host concentration (or count, as the distinction is arbitrary here) was initialized at 10^9^, antibody concentrations were proportional to the ones found using the oFLD-A protocol and scaled appropriately depending on the host concentration, $N_{o}$ was chosen to be 5, $N_{\text{ent}}$ was set to 2 for computational tractability, $k_{\text{rep}}$ was chosen to be 10^−11^, and the simulation time within each broth was $\Delta t_{b}=3\times 10^{9}$. Binding dynamics needed to be faster than replication dynamics; since only dissociation constants $K_{d}=k_{\text{off}}/k_{\text{on}}$ were prescribed by the force field calculations, we chose $k_{\text{on}}=10^{6}$ with $k_{\text{off}}=K_{d}\times k_{\text{on}}$. Our chosen $k_{\text{rep}}$ was appropriately much smaller than the binding rate constants.

For the first broth, viral concentrations were each initialized at 5,000. After running the BioNetGen ODE simulator for $\Delta t_{b}$, we computed viral concentrations and rounded them to the nearest integer (to approximately discretize non-integer concentrations to a viral count). We then sampled 10,000 virions, without replacement, to initialize the next broth. These viral counts, divided by the total count of 10,000, were recorded in the strain frequency timeseries. A maximum number of 50 broths were simulated for each test sequence, with broth transfers ending early if the either of the viral counts dropped below 100.

Given test strain frequency p(t) as a function of broth dilution index t and a total number of dilutions $t_{\text{max}}$, the fitness difference $F\left(s_{\text{test}}\right)-F\left(s_{\text{reference}}\right)$ was estimated from the strain frequency timeseries with

(M13) $$F\left(s_{\text{test}}\right)-F\left(s_{\text{reference}}\right)\approx \frac{1}{t_{max}\Delta t_{b}}\frac{p\left(t_{max}\right)-p(0)}{\langle p(t)\rangle -\left\langle p(t{)}^{2}\right\rangle },$$

where ⟨·⟩ is a time average. This is derived in Supplementary Note 3 from the 1-dimensional Kimura equation^1^, which is the diffusion limit of population genetics.

### 4.6 Iterative fitness landscape design with antibodies (iFLD-A) protocol

The iFLD-A protocol first finds an antibody which binds with high affinity to the starting strain (the WTT antibody). This is accomplished by using simulated annealing as used in the oFLD-A protocol, but with the following main modifications: (1) only a single antibody is used, (2) the antibody is at fixed concentration, so the concentration update steps are irrelevant, and (3) the loss function is set to the binding free energy $\Delta {\tilde{G}}_{Ab}^{\text{Potts}}(s,a)$. The simulation temperatures are $T_{MCMC}=0.1$ followed by 0.01. The simulated annealing generates the WTT antibody, which we then use to calculate the full fitness landscape over all antigenic sequences. By exhaustively searching the antigenic sequence space of 20^4^ = 160,000 variants (all amino acid sequences at the 4 antigenic sites) and using the biophysical model in eq. (1) to compute fitness, we identify the peak fitness and its antigenic sequence, which is set as the target sequence, and we once again run simulated annealing to find a high affinity antibody. A total of 5 iterations after finding the WTT antibody are performed.

For the cases where we used random starting sequences instead of the true SARS-CoV-2 wildtype, we performed a total of 200 random trials. 20 trials had a starting random (normalized) fitness between 0 and 0.1, 20 trials had starting fitness bewteen 0.1 and 0.2, and so forth. We averaged over all 200 trials to produce Figure 5D.

### Wright-Fisher viral evolutionary dynamics.

Wright-Fisher simulations follow the protocol in Greenbury et al.^5^, where we initialize $N_{\text{pop}}=1000$ antigen sequences at the SARS-CoV-2 wildtype. At each of the 10,000 time steps, we calculate all fitnesses for every individual in the population using eq. (M7). The next generation of 10,000 individuals is selected by sampling the previous generation’s individuals with replacement, with probability $p_{i}=F_{i}/\sum_{j=1}^{10,000}F_{j}$, where i indexes the previous generation’s individuals. Each of the new generation’s sequence sites are chosen for possible mutation with probability $\mu_{\text{chosen}}=0.01$ (for the polymorphic simulations) or $\mu_{\text{chosen}}=0.0001$ (for the near-monomorphic simulations). If a site is chosen to mutate, a random amino acid is chosen for that site. This makes the mutation probability $\mu =\mu_{\text{chosen}}\times 19/20$. The next time step then begins with fitness calculations of all sequences, which are stored. Since for each antigen we consider $L_{Ag}=4$ residues, the polymorphic dynamics have $N_{\text{pop}}\mu L_{Ag}=38$, and the near-monomorphic dynamics have $N_{\text{pop}}\mu L_{Ag}=0.38$.

## Supplementary Material

Supplement 1

## Data Availability

Code and data will be made publicly available upon publication.
