# Supplementary material for: Biophysical fitness landscape design traps viral evolution
Source: bioRxiv. 2025 Jul 25:2025.03.30.646233. Originally published 2025 Mar 31. Preprint. [Version 3] doi: 10.1101/2025.03.30.646233 (PMC11996392; doi:10.1101/2025.03.30.646233)
Supplement: Supplement 1 [file NIHPP2025.03.30.646233v3-supplement-1.pdf]

bioRxiv preprint doi: https://doi.org/10.1101/2025.03.30.646233; this version posted July 25, 2025. The copyright holder for this preprint (which was not certified by peer review) is the author/funder, who has granted bioRxiv a license to display the preprint in perpetuity. It is made available under aCC-BY 4.0 International license.

Supplementary Information

Supplementary Table

Molecule	Mutable Residues
SARS-CoV-2 Spike Protein	E484, G485, F486, Q493
Ly-CoV555 Light Chain	Y32, S91, Y92, S93, T94
Ly-CoV555 Heavy Chain	Y101, E102, A103, R104, Y109, Y110

Table S1: Mutable antigen and antibody residues used for fitness landscape design.

bioRxiv preprint doi: https://doi.org/10.1101/2025.03.30.646233; this version posted July 25, 2025. The copyright holder for this preprint (which was not certified by peer review) is the author/funder, who has granted bioRxiv a license to display the preprint in perpetuity. It is made available under aCC-BY 4.0 International license.

Supplementary Notes

Supplementary Note 1: *Binding and replication differential equations map onto absolute fitness.*

Binding reactions. We write down the definition of the dissociation constant for eq. (M2):

$$K_{d,\mathrm{H}}(\mathbf{s}) = \frac{[\mathrm{V_{ent}}(\mathbf{s})][\mathrm{H}]}{C_0[\mathrm{V_{ent}}(\mathbf{s})\cdot\mathrm{H}]}, \tag{S1}$$

and for eq. (M3)

$$K_{d,\mathrm{Ab}}(\mathbf{s},\mathbf{a}_n) = \frac{[\mathrm{V_{ent}}(\mathbf{s})][\mathrm{Ab}_n(\mathbf{a}_n)]}{C_0[\mathrm{V_{ent}}(\mathbf{s})\cdot\mathrm{Ab_n}(\mathbf{a_n})]}., \tag{S2}$$

where C_0 is the reference concentration used to make the dissociation constants dimensionless. We seek an expression for $p_b(\mathbf{s})$, the equilibirum host-bound fraction of viral entry proteins:

$$\begin{aligned} p_b(\mathbf{s}) &\equiv \frac{[\mathrm{V_{ent}}(\mathbf{s})\cdot\mathrm{H}]}{[\mathrm{V_{ent}^{total}}(\mathbf{s})]} \\ &= \frac{[\mathrm{V_{ent}}(\mathbf{s})\cdot\mathrm{H}]}{[\mathrm{V_{ent}}(\mathbf{s})] + [\mathrm{V_{ent}}(\mathbf{s})\cdot\mathrm{H}] + [\mathrm{V_{ent}}(\mathbf{s})\cdot\mathrm{Ab_n}(\mathbf{a_n})]}. \end{aligned} \tag{S3}$$

Substituting using eq. (S1) and eq. (S2), we obtain:

$$\begin{aligned} p_b(\mathbf{s}) &= \frac{\frac{[\mathrm{V_{ent}}(\mathbf{s})][\mathrm{H}]}{C_0K_{d,\mathrm{H}}(\mathbf{s})}}{[\mathrm{V_{ent}}(\mathbf{s})] + \frac{[\mathrm{V_{ent}}(\mathbf{s})][\mathrm{H}]}{C_0K_{d,\mathrm{H}}(\mathbf{s})} + \frac{[\mathrm{V_{ent}}(\mathbf{s})][\mathrm{Ab}_n(\mathbf{a}_n)]}{C_0K_{d,\mathrm{Ab}}(\mathbf{s},\mathbf{a}_n)}} \\ &= \frac{\frac{[\mathrm{H}]}{K_{d,\mathrm{H}}(\mathbf{s})}}{C_0 + \frac{[\mathrm{H}]}{K_{d,\mathrm{H}}(\mathbf{s})} + \frac{[\mathrm{Ab}_n(\mathbf{a}_n)]}{K_{d,\mathrm{Ab}}(\mathbf{s},\mathbf{a}_n)}} \end{aligned} \tag{S4}$$

Using $\Delta G_\mathrm{H}(\mathbf{s}) = \beta^{-1}\log K_{d,\mathrm{H}}(\mathbf{s})$ as well as $\Delta G_\mathrm{Ab}(\mathbf{s},\mathbf{a}_n) = \beta^{-1}\log K_{d,\mathrm{Ab}}(\mathbf{s},\mathbf{a}_n)$, we arrive at

$$p_b(\mathbf{s}) = \frac{[\mathrm{H}]e^{-\beta\Delta G_\mathrm{Ab}(\mathbf{s})}}{C_0 + [\mathrm{H}]e^{-\beta\Delta G_\mathrm{H}(\mathbf{s})} + \sum_n[\mathrm{Ab}_n(\mathbf{a}_n)]e^{-\beta\Delta G_\mathrm{Ab}(\mathbf{s},\mathbf{a}_n)}}. \tag{S5}$$

$[\mathrm{H}]$ and $[\mathrm{Ab}_n(\mathbf{a}_n)]$ are unbound host and antibody concentrations.

We assume that the availability of infectable host cells and receptors is much higher than the viral load, and clinical data suggests that antibody concentrations are much higher than the viral concentration in a immunocompetent, vaccinated individual (Supplementary Note 2). Assuming that host receptors and antibodies are not significantly depleted by viral binding, we can approximately equate the free and experimentally controllable total concentrations $[\mathrm{H^{total}}] \approx [\mathrm{H}]$ and $[\mathrm{Ab}_n^\mathrm{total}(\mathbf{a}_n)] \approx [\mathrm{Ab}_n(\mathbf{a}_n)]$. Thus, we have

$$p_b(\mathbf{s}) \approx \frac{[\mathrm{H^{total}}]e^{-\beta\Delta G_\mathrm{Ab}(\mathbf{s})}}{C_0 + [\mathrm{H^{total}}]e^{-\beta\Delta G_\mathrm{H}(\mathbf{s})} + \sum_n[\mathrm{Ab}_n^\mathrm{total}(\mathbf{a}_n)]e^{-\beta\Delta G_\mathrm{Ab}(\mathbf{s},\mathbf{a}_n)}}, \tag{S6}$$

which is eq. (2).

bioRxiv preprint doi: https://doi.org/10.1101/2025.03.30.646233; this version posted July 25, 2025. The copyright holder for this preprint (which was not certified by peer review) is the author/funder, who has granted bioRxiv a license to display the preprint in perpetuity. It is made available under aCC-BY 4.0 International license.

Replication dynamics. We note that the chemical reaction eq. (S7) produces *unbound* copies of the virus, which we temporarily denote as $V(\mathbf{s})\cdot(0\text{ H})$ for mathematical convenience. Thus, we have

$$\mathrm{V}(\mathbf{s})\cdot(j\,\mathrm{H}) \xrightarrow{j\,k_{\mathrm{rep}}} N_o\,\mathrm{V}(\mathbf{s})\cdot(0\,\mathrm{H}). \tag{S7}$$

The ordinary differential equation for the time evolution of the unbound concentration must include contributions from the rate equations for values of all $j \in \{1,\dots,N_{\mathrm{ent}}\}$:

$$\frac{\mathrm{d}[\mathrm{V}(\mathbf{s})\cdot(0\ \mathrm{H})]}{\mathrm{d}t} = N_o \sum_{j=1}^{N_{\mathrm{ent}}} (jk_{\mathrm{rep}})[\mathrm{V}(\mathbf{s})\cdot(j\ \mathrm{H})]. \tag{S8}$$

The same reaction equation also provides the depletion rate of the replicating virus bound to j host receptors:

$$\frac{\mathrm{d}[\mathrm{V}(\mathbf{s})\cdot(j\ \mathrm{H})]}{\mathrm{d}t} = -(jk_{\mathrm{rep}})[\mathrm{V}(\mathbf{s})\cdot(j\ \mathrm{H})], \quad 1 \le j \le N_{\mathrm{ent}} \tag{S9}$$

We now denote the total viral concentration for strain $\mathbf{s}$ as

$$[\mathrm{V}^{\mathrm{tot}}(\mathbf{s})] = \sum_{j=0}^{N_{\mathrm{ent}}} [\mathrm{V}(\mathbf{s})\cdot(j\ \mathrm{H})], \tag{S10}$$

which includes all viruses in all bound and unbound states. Taking a time derivative, it immediately follows that

$$\begin{aligned}\frac{\mathrm{d}[\mathrm{V}^{\mathrm{tot}}(\mathbf{s})]}{\mathrm{d}t} &= \sum_{j=0}^{N_{\mathrm{ent}}} \frac{\mathrm{d}[\mathrm{V}(\mathbf{s})\cdot(j\ \mathrm{H})]}{\mathrm{d}t} \\ &= \frac{\mathrm{d}[\mathrm{V}(\mathbf{s})\cdot(0\ \mathrm{H})]}{\mathrm{d}t} + \sum_{j=1}^{N_{\mathrm{ent}}} \frac{\mathrm{d}[\mathrm{V}(\mathbf{s})\cdot(j\ \mathrm{H})]}{\mathrm{d}t}.\end{aligned} \tag{S11}$$

Plugging in the results of eq. (S8) and eq. (S9), we immediately have

$$\begin{aligned}\frac{\mathrm{d}[\mathrm{V}^{\mathrm{tot}}(\mathbf{s})]}{\mathrm{d}t} &= N_o \sum_{j=1}^{N_{\mathrm{ent}}} (jk_{\mathrm{rep}})[\mathrm{V}(\mathbf{s})\cdot(j\ \mathrm{H})] - \sum_{j=1}^{N_{\mathrm{ent}}} (jk_{\mathrm{rep}})[\mathrm{V}(\mathbf{s})\cdot(j\ \mathrm{H})] \\ &= (N_o - 1)k_{\mathrm{rep}} \sum_{j=1}^{N_{\mathrm{ent}}} j[\mathrm{V}(\mathbf{s})\cdot(j\ \mathrm{H})].\end{aligned} \tag{S12}$$

We now invoke separation of timescales: if the binding dynamics occur at timescales much faster than replication, then $[\mathrm{V}(\mathbf{s})\cdot(j\ \mathrm{H})]$ can be calculated at equilibrium. At equilibrium, we showed that the probability of finding any viral entry protein bound to a host receptor is $p_b(\mathbf{s})$. Thus, the number of entry proteins on a single virion which are bound to host receptors is a binomial random variable $J \sim \mathrm{Binomial}(N_{\mathrm{ent}}, p_b(\mathbf{s}))$. The fraction of virions bound to j hosts should follow the distribution of J:

$$[\mathrm{V}(\mathbf{s})\cdot(j\ \mathrm{H})] = [\mathrm{V}^{\mathrm{tot}}(\mathbf{s})]\mathbb{P}[J = j] \tag{S13}$$

bioRxiv preprint doi: https://doi.org/10.1101/2025.03.30.646233; this version posted July 25, 2025. The copyright holder for this preprint (which was not certified by peer review) is the author/funder, who has granted bioRxiv a license to display the preprint in perpetuity. It is made available under aCC-BY 4.0 International license.

Substituting into eq. (S12), it follows that

$$\begin{aligned}\frac{\mathrm{d}[\mathrm{V}^{\mathrm{tot}}(\mathbf{s})]}{\mathrm{d}t} &= (N_o - 1)k_{\mathrm{rep}}[\mathrm{V}^{\mathrm{tot}}(\mathbf{s})]\sum_{j=1}^{N_{\mathrm{ent}}} j\mathbb{P}[J = j] \\ &= (N_o - 1)k_{\mathrm{rep}}[\mathrm{V}^{\mathrm{tot}}(\mathbf{s})]\sum_{j=0}^{N_{\mathrm{ent}}} j\mathbb{P}[J = j].\end{aligned} \tag{S14}$$

Noting that the sum is exactly the expectation of a binomial variable, we have

$$\sum_{j=0}^{N_{\mathrm{ent}}} j\mathbb{P}[J = j] = \mathbb{E}[J] = N_{\mathrm{ent}}p_b(\mathbf{s}), \tag{S15}$$

which provides the final result

$$\frac{\mathrm{d}[\mathrm{V}^{\mathrm{tot}}(\mathbf{s})]}{\mathrm{d}t} = (N_o - 1)k_{\mathrm{rep}}N_{\mathrm{ent}}p_b(\mathbf{s})[\mathrm{V}^{\mathrm{tot}}(\mathbf{s})], \tag{S16}$$

which is eq. (M5), the desired result.

bioRxiv preprint doi: https://doi.org/10.1101/2025.03.30.646233; this version posted July 25, 2025. The copyright holder for this preprint (which was not certified by peer review) is the author/funder, who has granted bioRxiv a license to display the preprint in perpetuity. It is made available under aCC-BY 4.0 International license.

Supplementary Note 2: *Justficiation of antibody and viral receptor relative concentrations from clinical data.* We note that a 50-year-old's mean peak viral load from a SARS-CoV-2 infection—independent of vaccination status—is $10^{8.14} \approx 1.28 \times 10^8$ copies/mL[64]. Each SARS-CoV-2 virion has, on average, 24 ± 9 spike protein trimers, so there are on average $72 = 7.2 \times 10^1$ spike proteins per virion[65]. Together, this suggests that an infected individual has $[\mathrm{V}_{\mathrm{ent}}(\mathbf{s})] \approx (1.28 \times 10^8) \times (7.2 \times 10^1) \approx 9.22 \times 10^9$ proteins/mL.

Recent clinical data[66] shows that previously uninfected individuals who received 2 doses of the SARS-CoV-2 mRNA vaccine had median spike protein-specific IgG concentration of 46.80 μg/mL, with previously infected but unvaccinated invidiuals having roughly 1 order of magnitude lower concentration and vaccinated individuals who had also been previously infected having roughly 1 order of magnitude greater concentration. Since IgGs have an average molecular weight of 150 kDa (kg/mol)[67], we approximately have

$$\frac{46.8\ \mu\mathrm{g}}{1\ \mathrm{mL}} \times \frac{1\ \mathrm{mol}}{150 \times 10^9\ \mu\mathrm{g}} \times \frac{6.02 \times 10^{23}\ \mathrm{antibodies}}{1\ \mathrm{mol}} = 1.88 \times 10^{14}\ \mathrm{antibodies/mL}, \tag{S17}$$

suggesting that antibody concentration outweighs viral concentration. Even with up to 10^2-10^3 orders of magnitude of epitope diversity, the estimated antibody concentration outweighs SARS-CoV-2 spike proteins.

The total host concentration is the sum of bound and unbound host concentrations $[\mathrm{H}^{\mathrm{total}}] = [\mathrm{H}] + [\mathrm{V}_{\mathrm{ent}}(\mathbf{s}) \cdot \mathrm{H}]$, and the total antibody concentration is the sum of bound and unbound host concentrations $[\mathrm{Ab}_n^{\mathrm{total}}(\mathbf{a}_n)] = [\mathrm{Ab}_n(\mathbf{a}_n)] + [\mathrm{V}_{\mathrm{ent}}(\mathbf{s}) \cdot \mathrm{Ab}_{\mathrm{n}}(\mathbf{a}_{\mathrm{n}})]$. Since we estimate antibody concentrations are greater than the number of viral proteins by orders of magnitude and assume vast availability of infectable host cells, we can write $[\mathrm{V}_{\mathrm{ent}}(\mathbf{s}) \cdot \mathrm{H}] < [\mathrm{V}_{\mathrm{ent}}^{\mathrm{total}}(\mathbf{s})] \ll [\mathrm{H}]$ and $[\mathrm{V}_{\mathrm{ent}}(\mathbf{s}) \cdot \mathrm{Ab}_{\mathrm{n}}(\mathbf{a}_{\mathrm{n}})] < [\mathrm{V}_{\mathrm{ent}}^{\mathrm{total}}(\mathbf{s})] \ll [\mathrm{Ab}_n^{\mathrm{total}}(\mathbf{a}_n)]$. Therefore, we can approximately equate the free and experimentally controllable total concentrations $[\mathrm{H}^{\mathrm{total}}] \approx [\mathrm{H}]$ and $[\mathrm{Ab}_n^{\mathrm{total}}(\mathbf{a}_n)] \approx [\mathrm{Ab}_n(\mathbf{a}_n)]$.

bioRxiv preprint doi: https://doi.org/10.1101/2025.03.30.646233; this version posted July 25, 2025. The copyright holder for this preprint (which was not certified by peer review) is the author/funder, who has granted bioRxiv a license to display the preprint in perpetuity. It is made available under aCC-BY 4.0 International license.

Supplementary Note 3: *Fitness estimation from strain frequency time series.* When there are two strains (test and reference), and $p(t)$ is the test strain frequency as a function of time t, the mean rate of change of the strain frequency—in continuous time—is given by the 1-dimensional Kimura equation[1] in the limit of no stochastic fluctuations (equivalently, the infinite population limit):

$$\frac{\partial p(t)}{\partial t} = p(t)(1-p(t))(F(\mathbf{s}_{\text{test}}) - F(\mathbf{s}_{\text{reference}})). \tag{S18}$$

First, we account for the fact that the *in silico* serial dilution experiment timescale is different from the broth dilution time scale. Any exponential growth during a time Δt_b in the simulation time looks like unit time in the broth time series. Thus, the fitnesses in the equation above need to be multiplied by a factor of Δt_b. Thus, we have

$$\frac{\partial p(t)}{\partial t} = \Delta t_b p(t)(1-p(t))F(\mathbf{s}_{\text{test}}) - F(\mathbf{s}_{\text{reference}}). \tag{S19}$$

Now, time averaging over both sides of the equation from $[0, t_{\text{max}}]$, we have

$$\begin{aligned} \frac{1}{t_{\text{max}}}\int_0^{t_{\text{max}}} \mathrm{d}t\, \frac{\partial p(t)}{\partial t} &= \frac{\Delta t_b}{t_{\text{max}}}\int_0^{t_{\text{max}}} \mathrm{d}t\, p(t)(1-p(t))\left(F(\mathbf{s}_{\text{test}}) - F(\mathbf{s}_{\text{reference}})\right) \\ \Rightarrow \frac{p(t_{\text{max}}) - p(0)}{t_{\text{max}}} &= \Delta t_b \left(\langle p(t)\rangle - \langle p(t)^2\rangle\right)\left(F(\mathbf{s}_{\text{test}}) - F(\mathbf{s}_{\text{reference}})\right), \end{aligned} \tag{S20}$$

which is rearranged to

$$F(\mathbf{s}_{\text{test}}) - F(\mathbf{s}_{\text{reference}}) \approx \frac{1}{t_{\text{max}}\Delta t_b}\frac{p(t_{\text{max}}) - p(0)}{\langle p(t)\rangle - \langle p(t)^2\rangle}. \tag{S21}$$

The same formula derived with forward-time finite difference will give time averages which are off by a boundary term in the series. We have not investigated the effect of deriving the above formula with an integral versus a sum but do not expect fitness estimation to be substantially affected.

bioRxiv preprint doi: https://doi.org/10.1101/2025.03.30.646233; this version posted July 25, 2025. The copyright holder for this preprint (which was not certified by peer review) is the author/funder, who has granted bioRxiv a license to display the preprint in perpetuity. It is made available under aCC-BY 4.0 International license.

Supplementary Figures

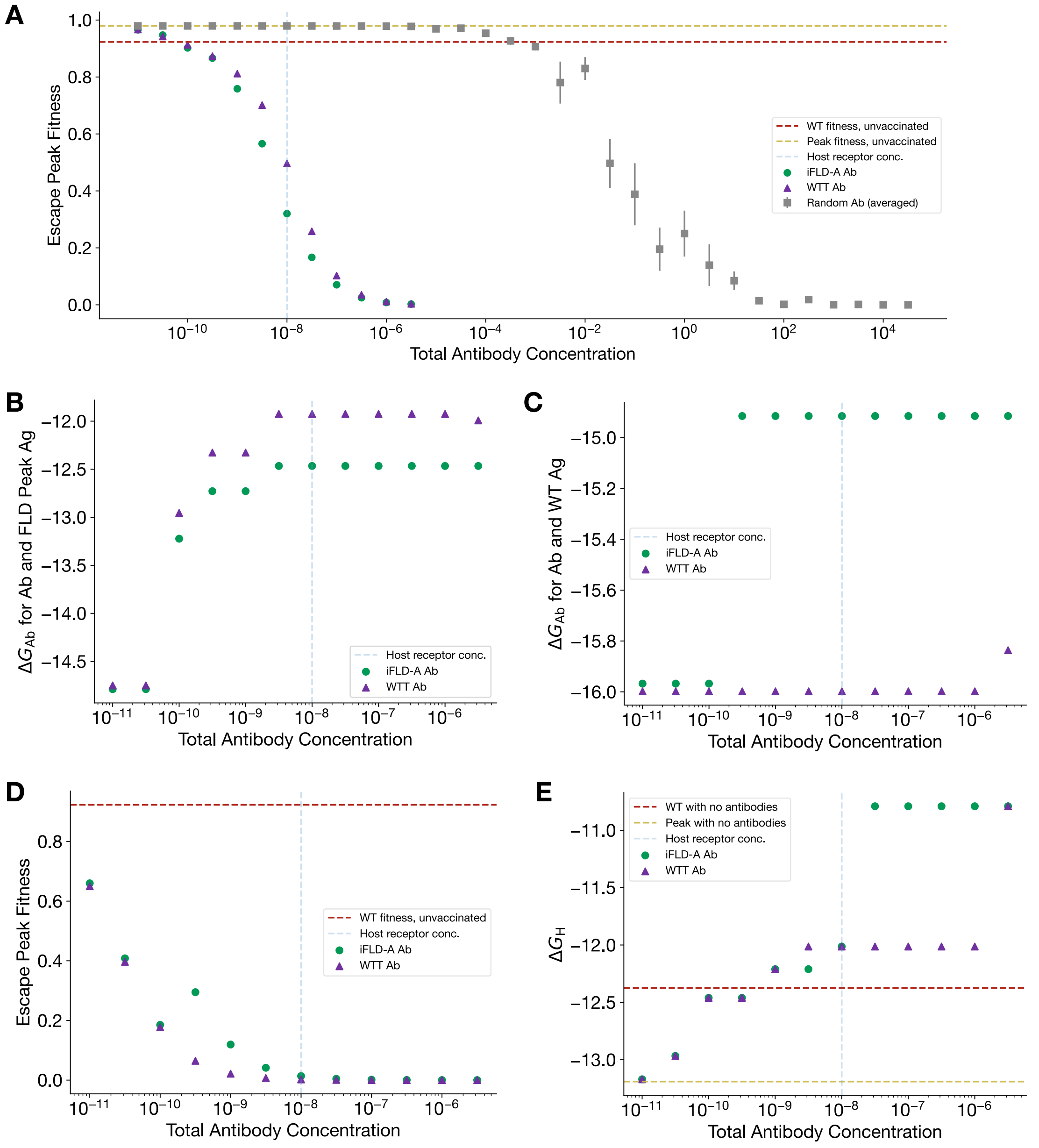

Figure S1: **Extended data for iFLD-A protocol with SARS-CoV-2 wildtype.** (**A**) Extended range of random antibody concentrations show fitness peak reduction is only possible at concentrations much higher than host concentration. (**B**) The iFLD-A antibody binds tighter to its target antigen than the WTT antibody does. (**C**) The iFLD-A antibody binds less strongly to the wildtype antigen than the WTT antibody does. (**D**) Despite weaker binding to the wildtype antigen, the iFLD-A still substantially suppresses wildtype fitness, often reducing it as much as the WTT antigen. (**E**) Both the iFLD-A antibody and the WTT antibody result in post-vaccination fitness peaks for which the highest-fitness escape variants bind the host receptor less strongly than in absence of any antibody.

bioRxiv preprint doi: https://doi.org/10.1101/2025.03.30.646233; this version posted July 25, 2025. The copyright holder for this preprint (which was not certified by peer review) is the author/funder, who has granted bioRxiv a license to display the preprint in perpetuity. It is made available under aCC-BY 4.0 International license.

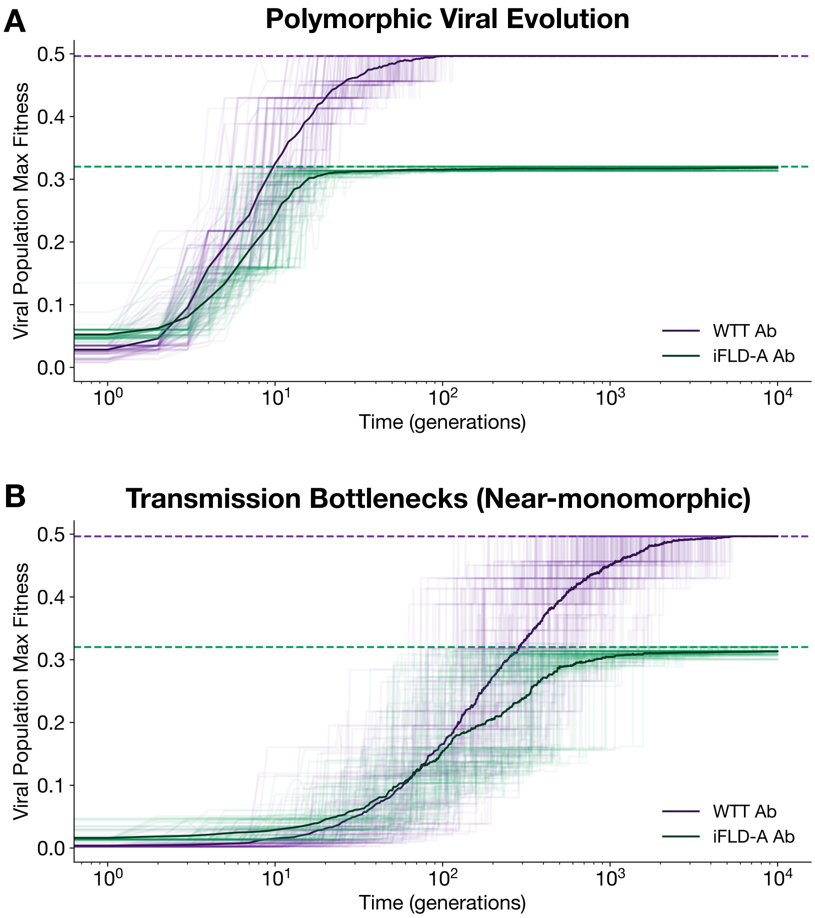

Figure S2: **Trajectories of population maximum fitness.** (**A**) Polymorphic and (**B**) near-monomorphic viral evolutionary dynamics, with trajectories corresponding to the highest fitness in the population at any given time. In both regimes, populations exhibit slower fitness growth and are trapped under a lower fitness ceiling by the iFLD-A antibody compared to the WTT antibody. Dashed lines indicate global maximum fitness, and translucent lines are population mean fitness trajectories from each of 100 Wright-Fisher trials.

bioRxiv preprint doi: https://doi.org/10.1101/2025.03.30.646233; this version posted July 25, 2025. The copyright holder for this preprint (which was not certified by peer review) is the author/funder, who has granted bioRxiv a license to display the preprint in perpetuity. It is made available under aCC-BY 4.0 International license.

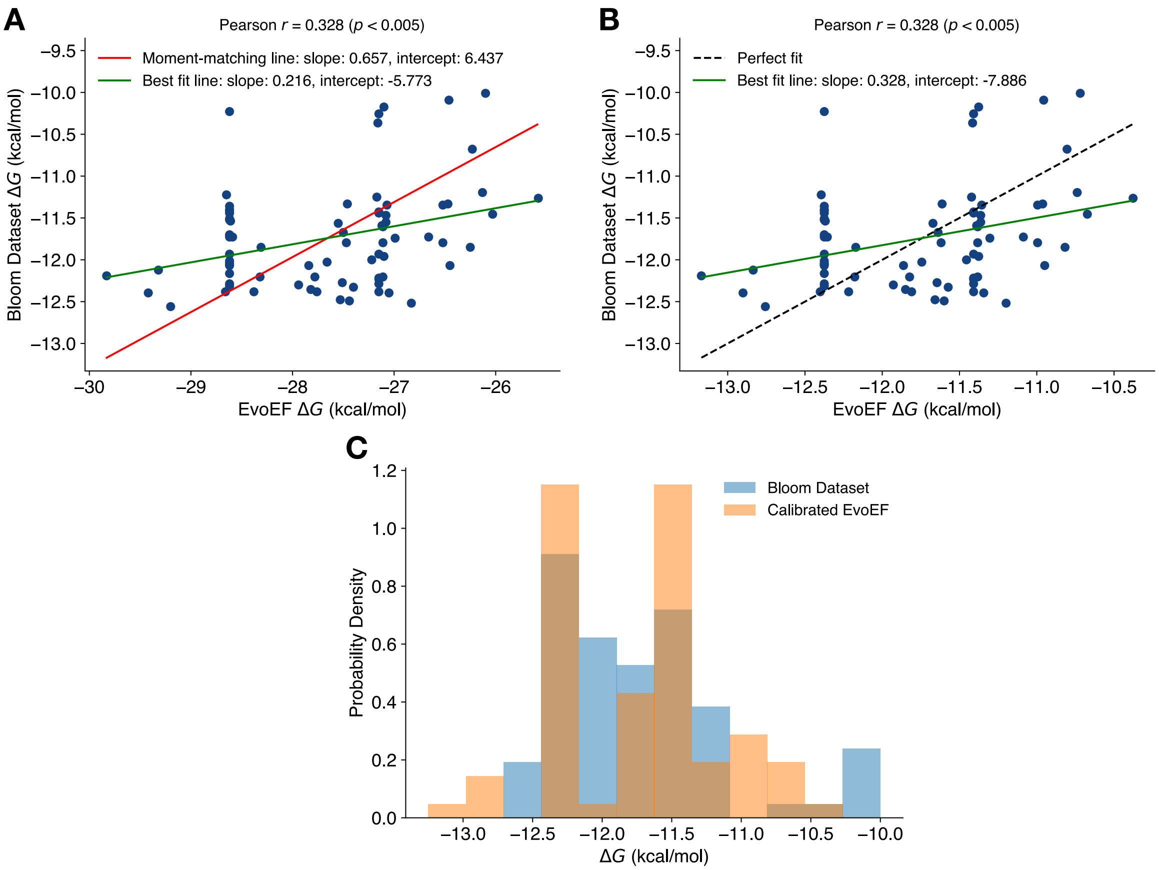

Figure S3: **Calibration of host-antigen force field binding affinities to experimental measurements**. (**A**) Force field and experimental binding affinities for the 77 antigens present in both datasets are positively correlated. (**B**) Calibration of force field binding affinities using moment-matching helps bring force field binding affinities absolutely closer to experimental ones. (**C**) The experimental data distribution and the calibrated force field data have matching means and variances.

bioRxiv preprint doi: https://doi.org/10.1101/2025.03.30.646233; this version posted July 25, 2025. The copyright holder for this preprint (which was not certified by peer review) is the author/funder, who has granted bioRxiv a license to display the preprint in perpetuity. It is made available under aCC-BY 4.0 International license.

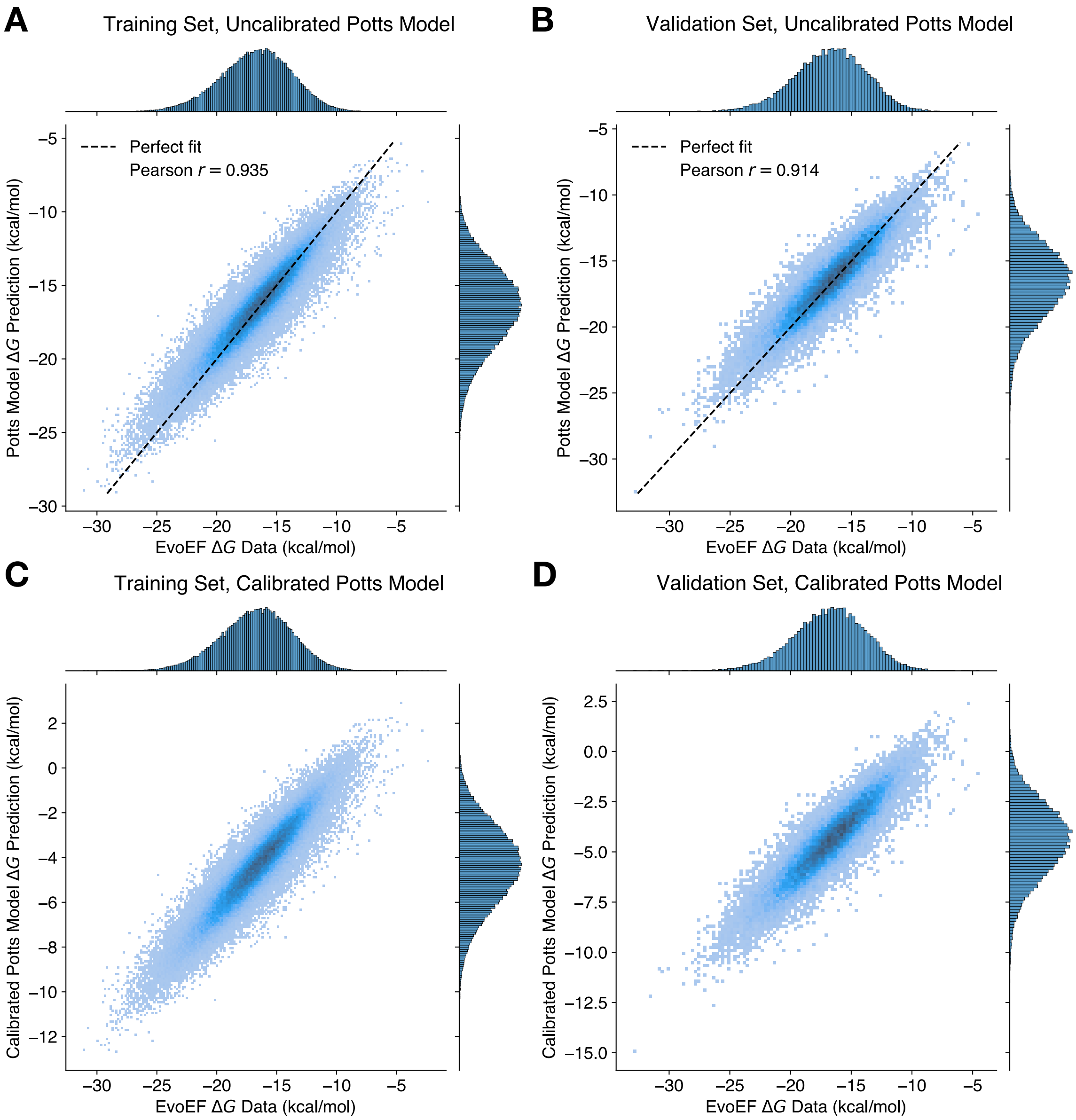

Figure S4: **Training, validation, and calibration (to experimental data) of Potts model for antibody-antigen binding affinities**. Potts model predictions on (**A**) training and (**B**) validation force field binding affinities show internal consistency. (**C**) and (**D**) show the same, but after calibration of the Potts model to experimental data.
